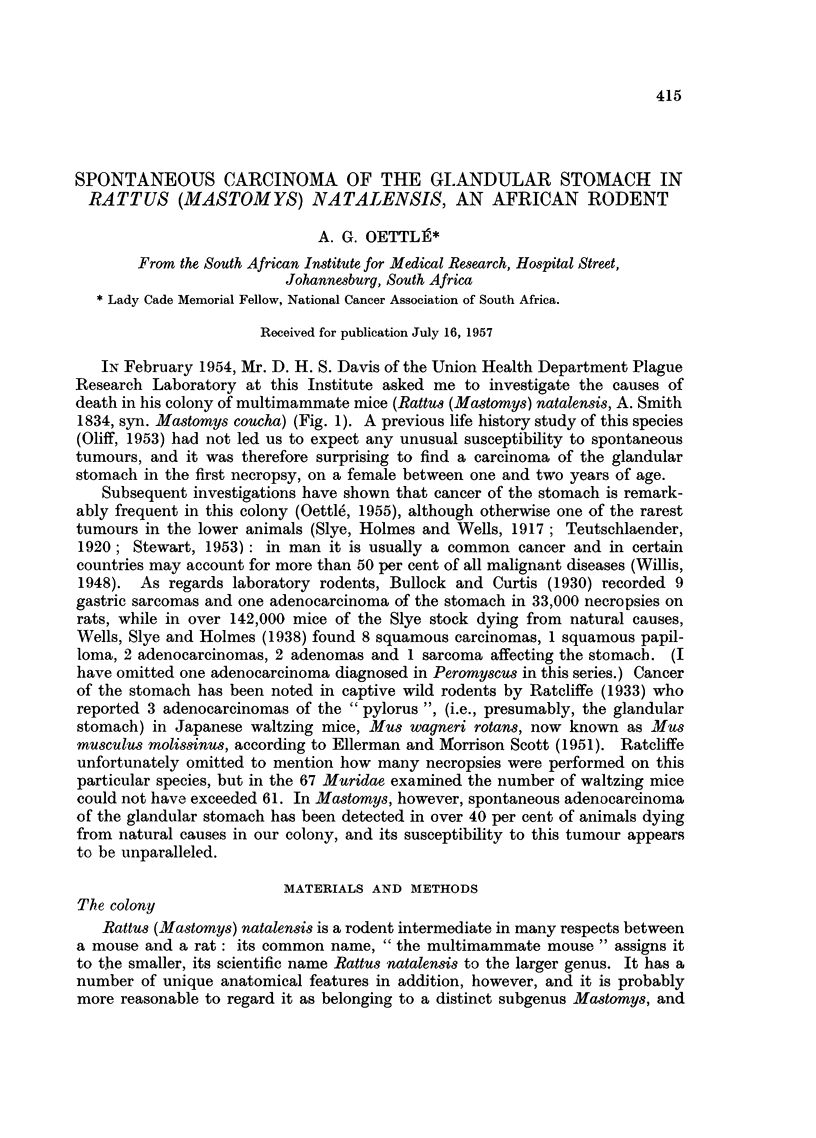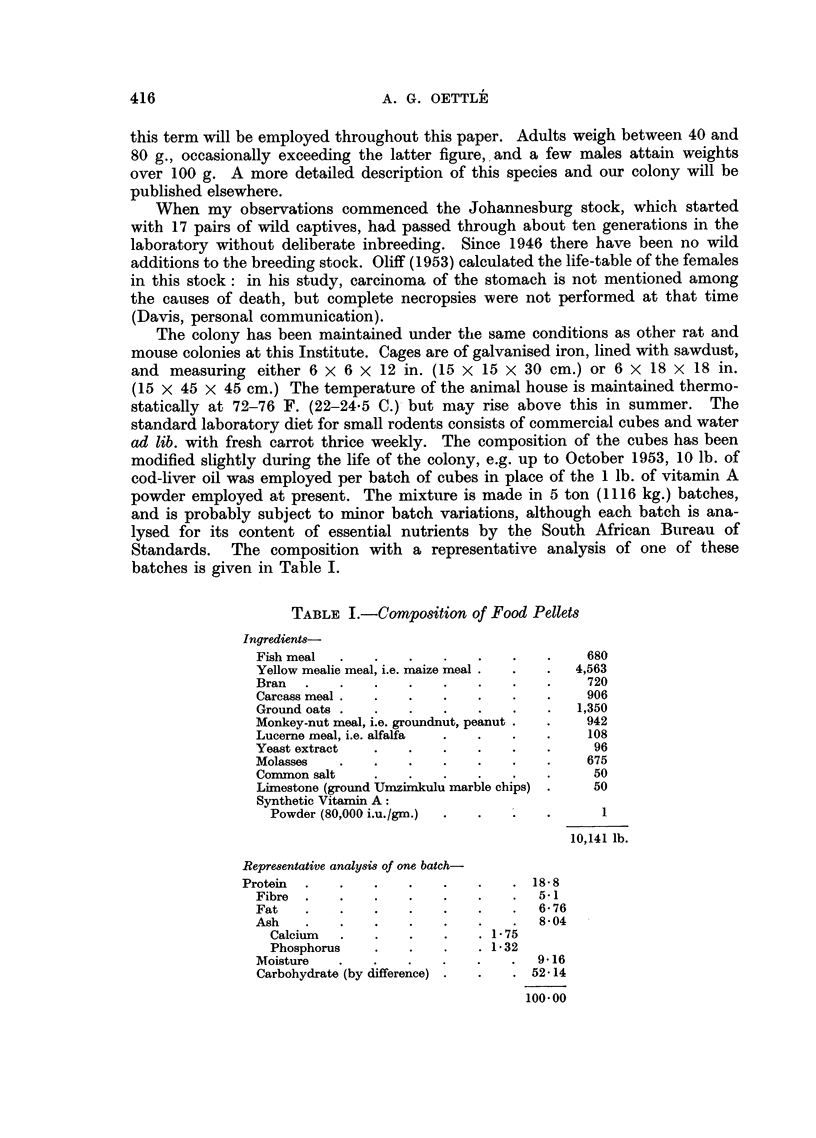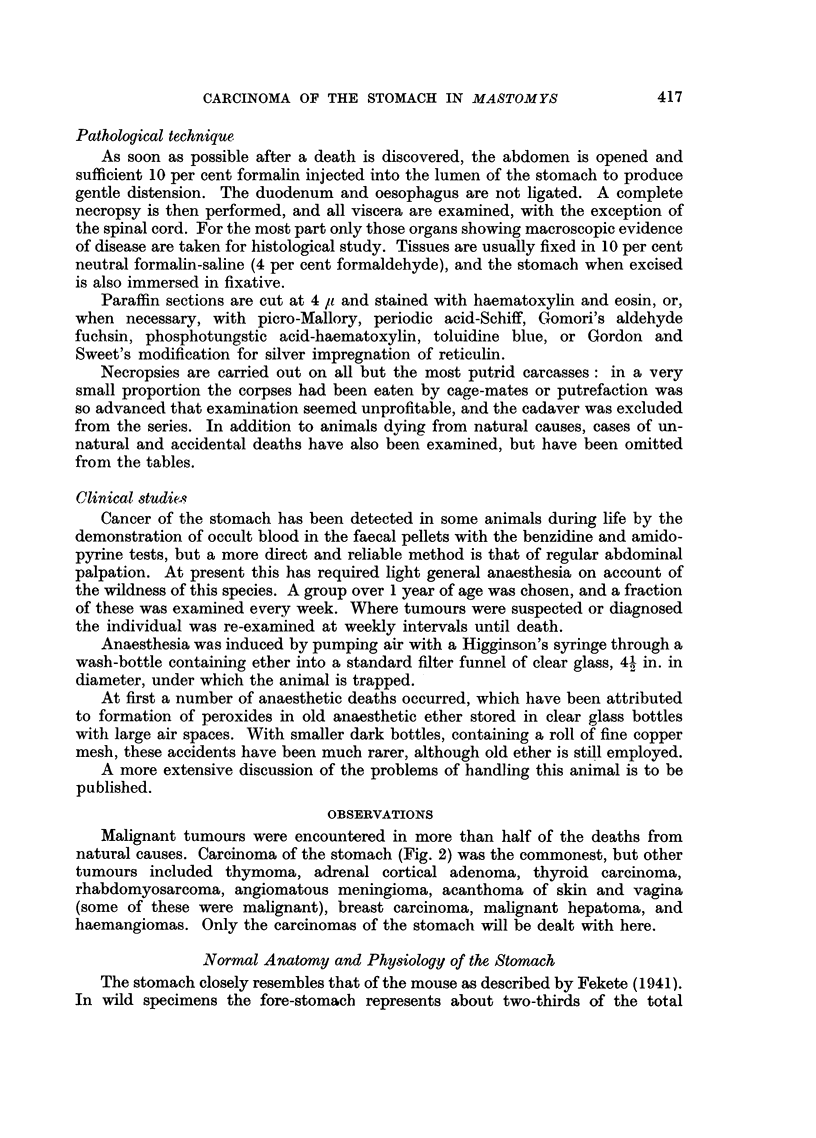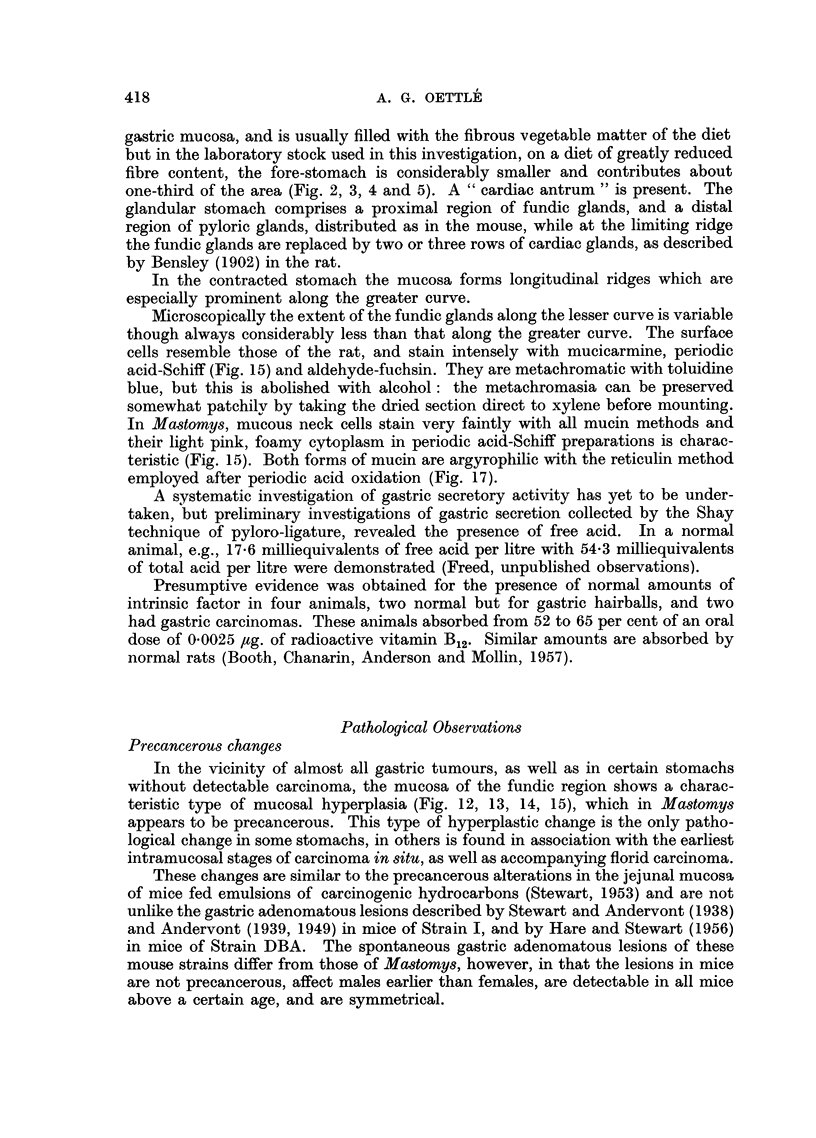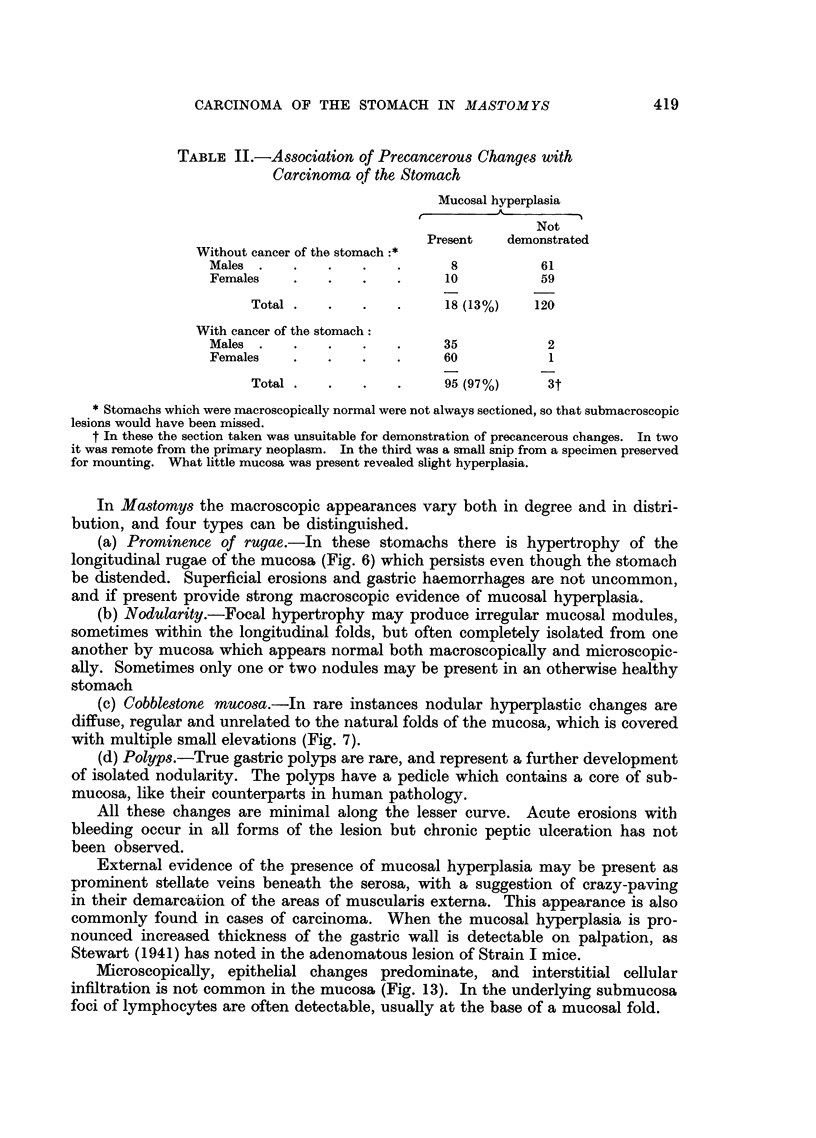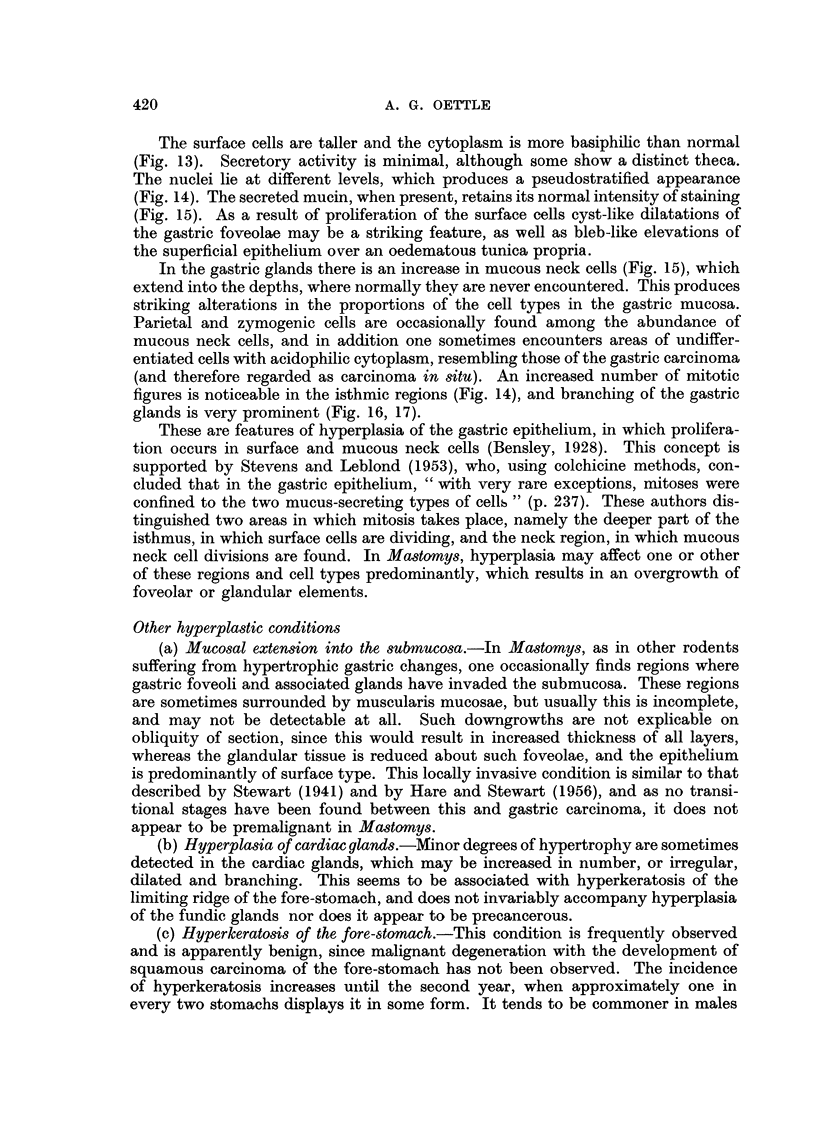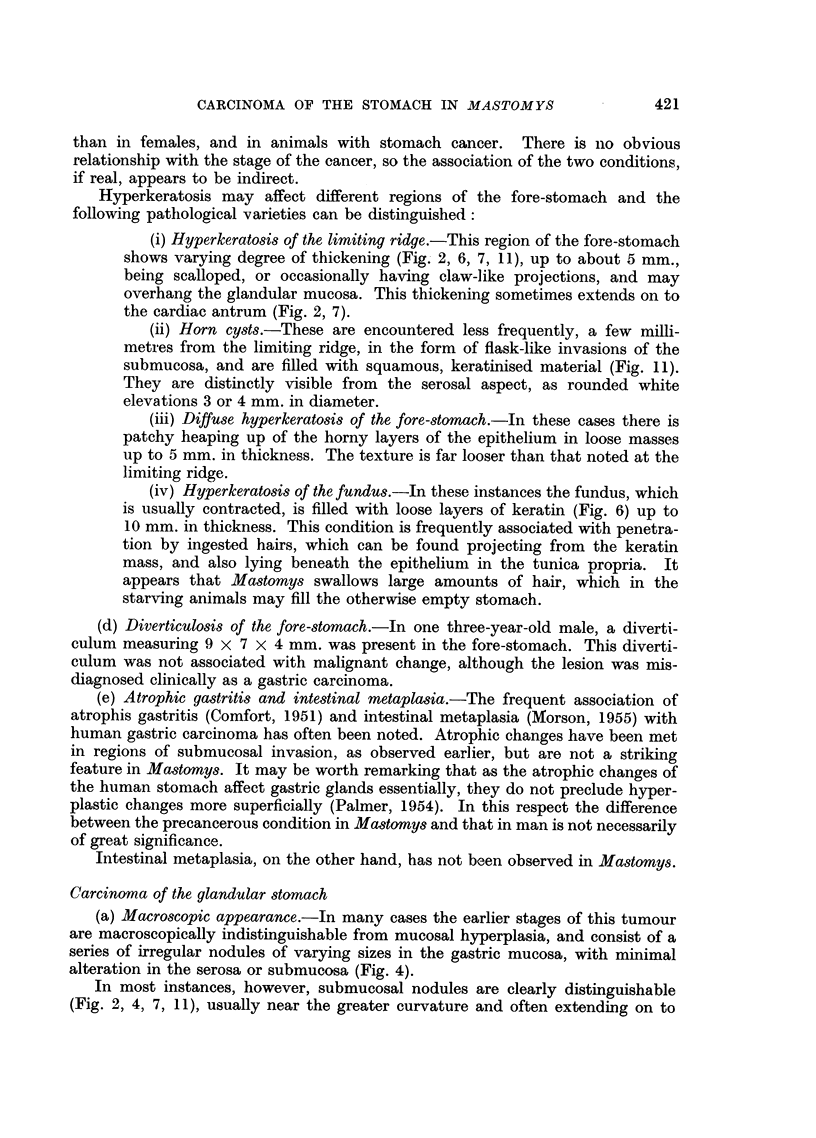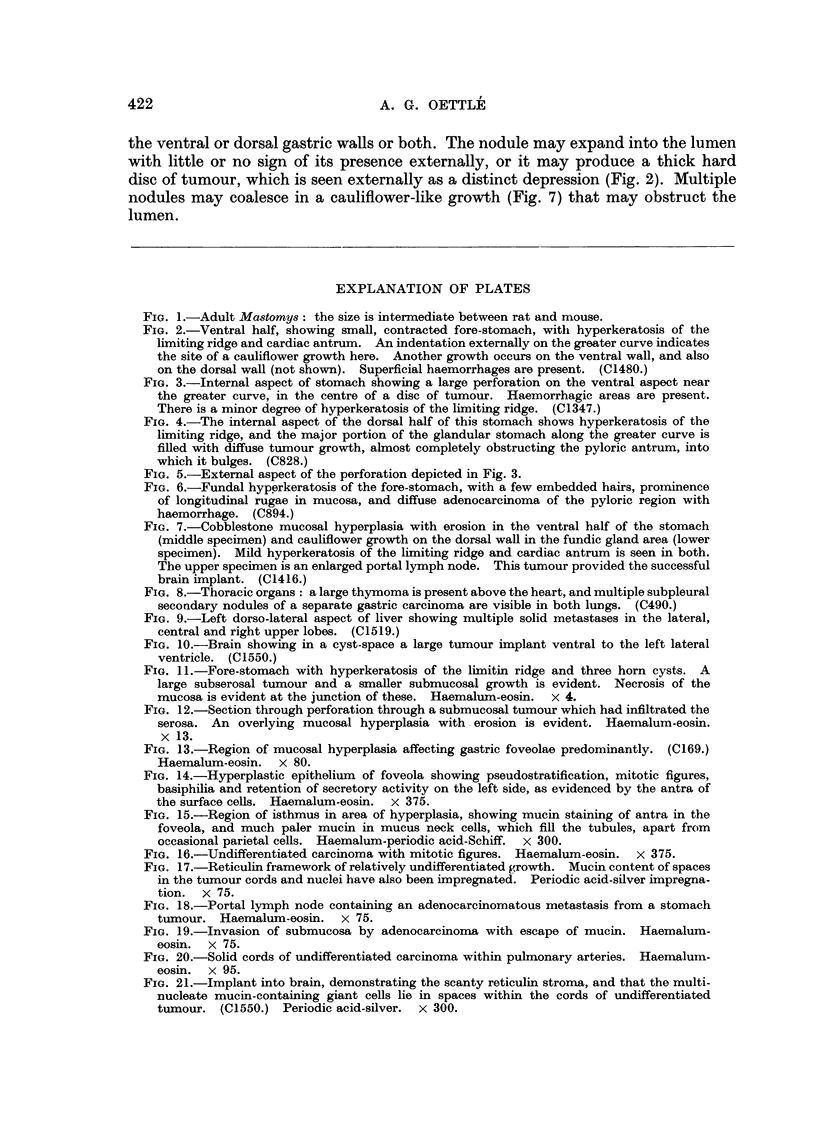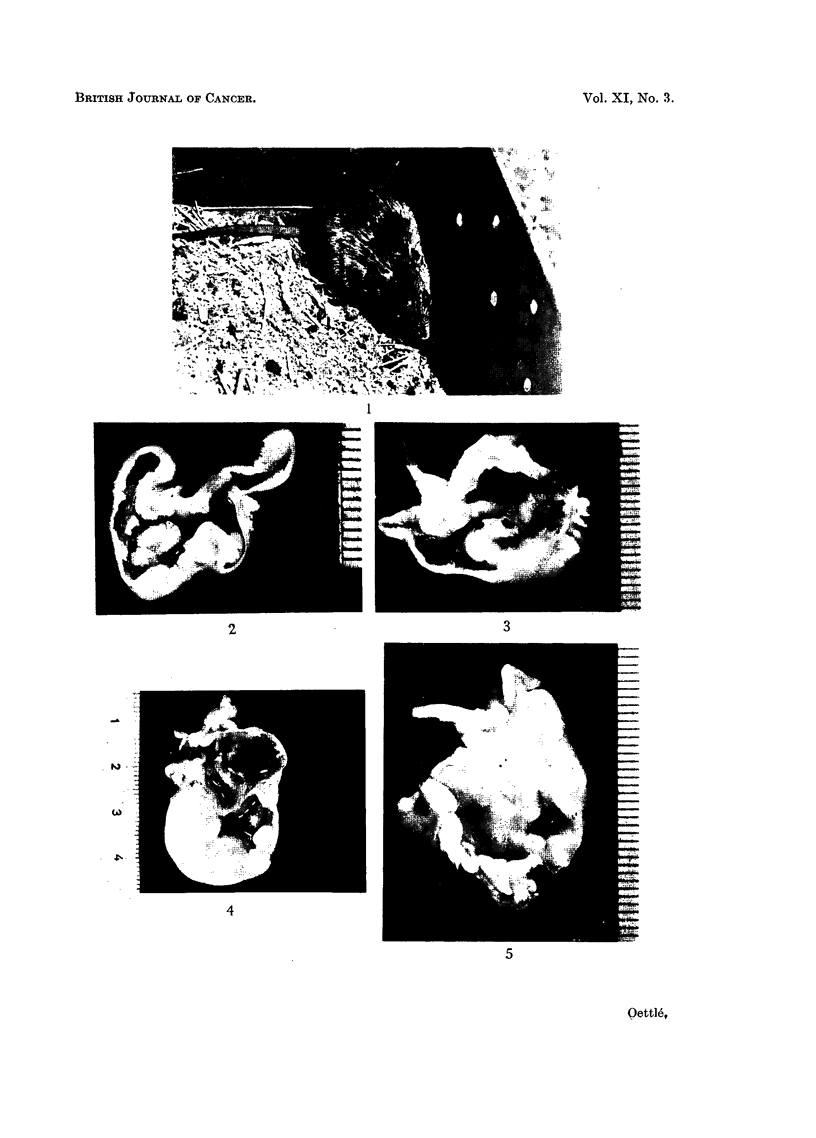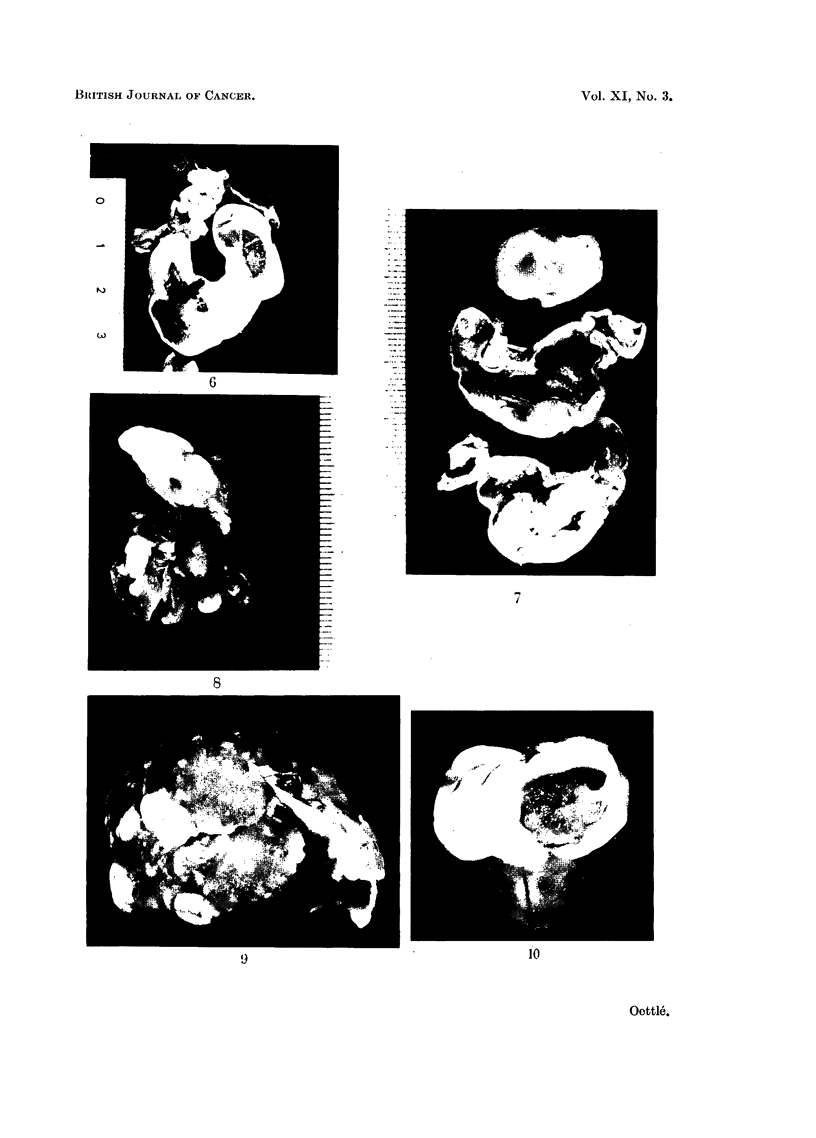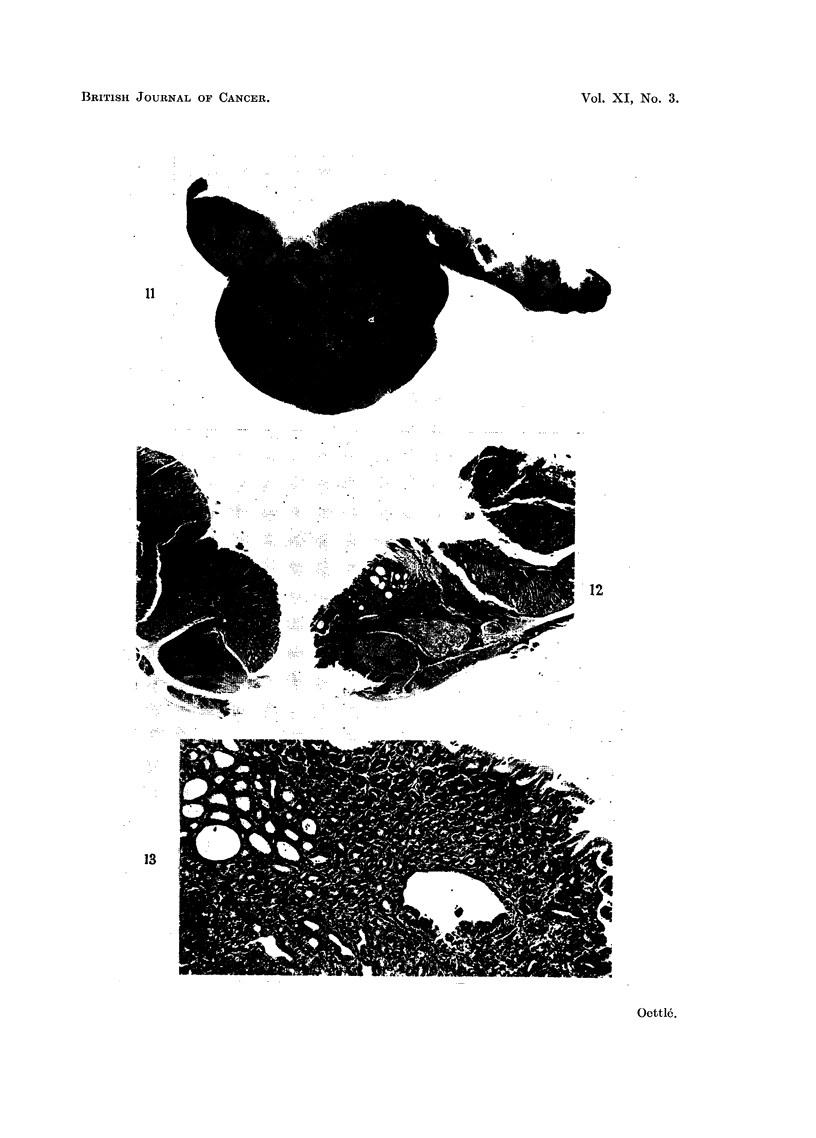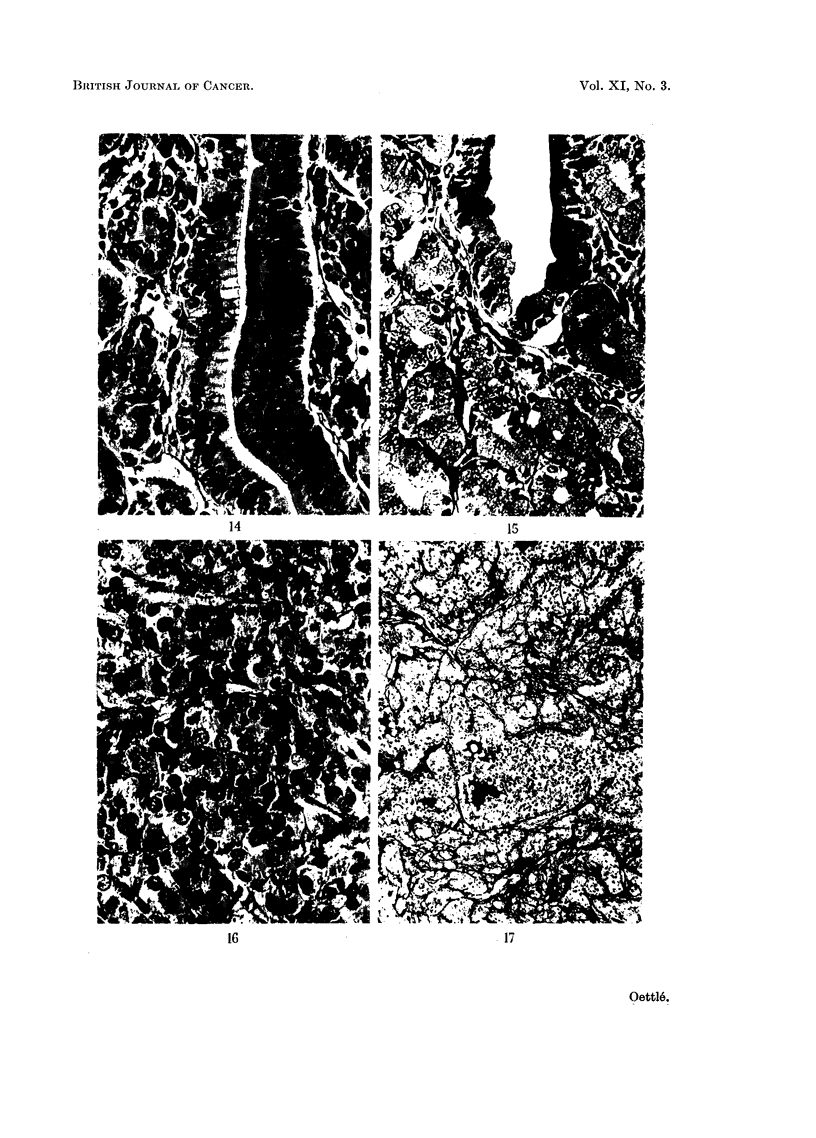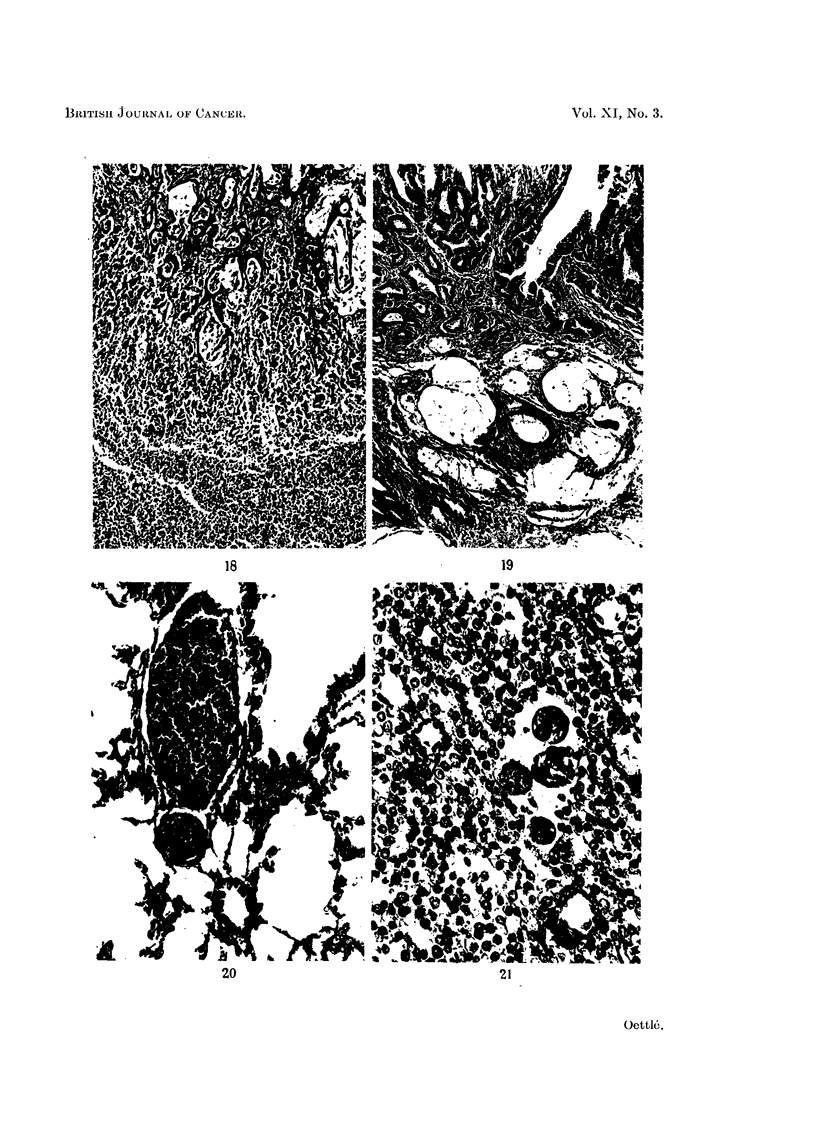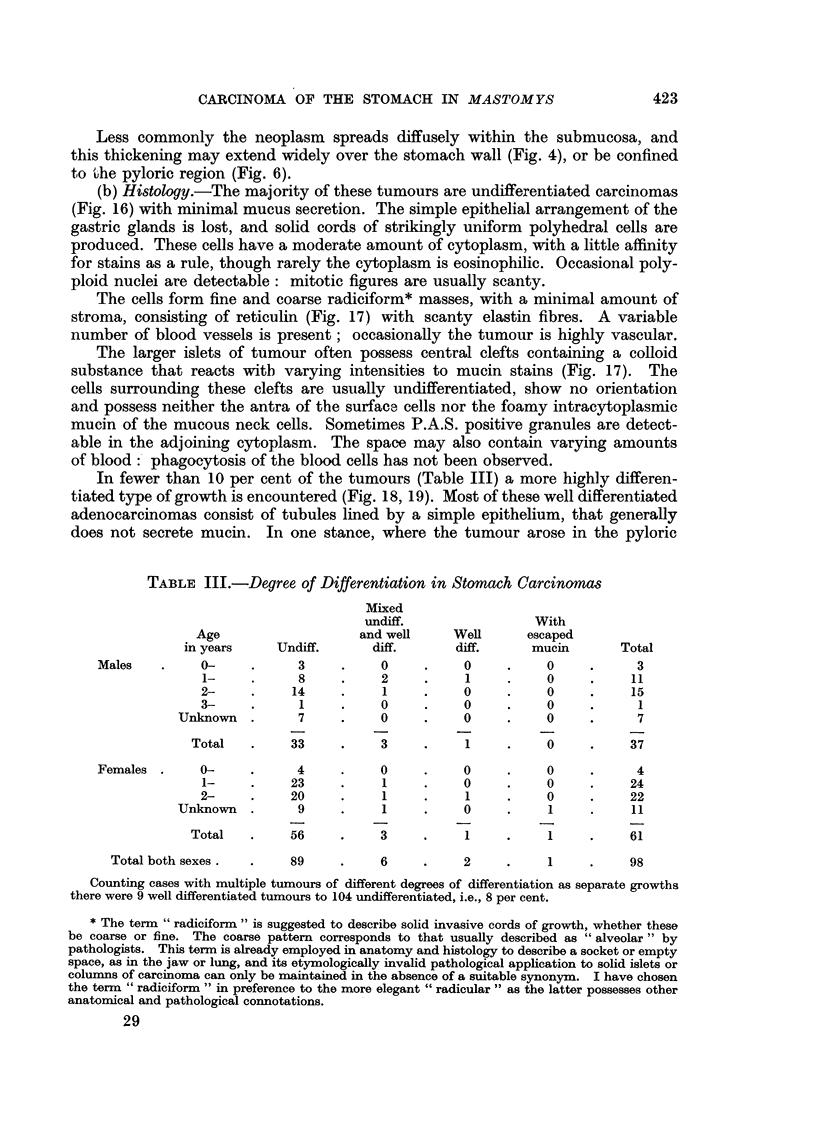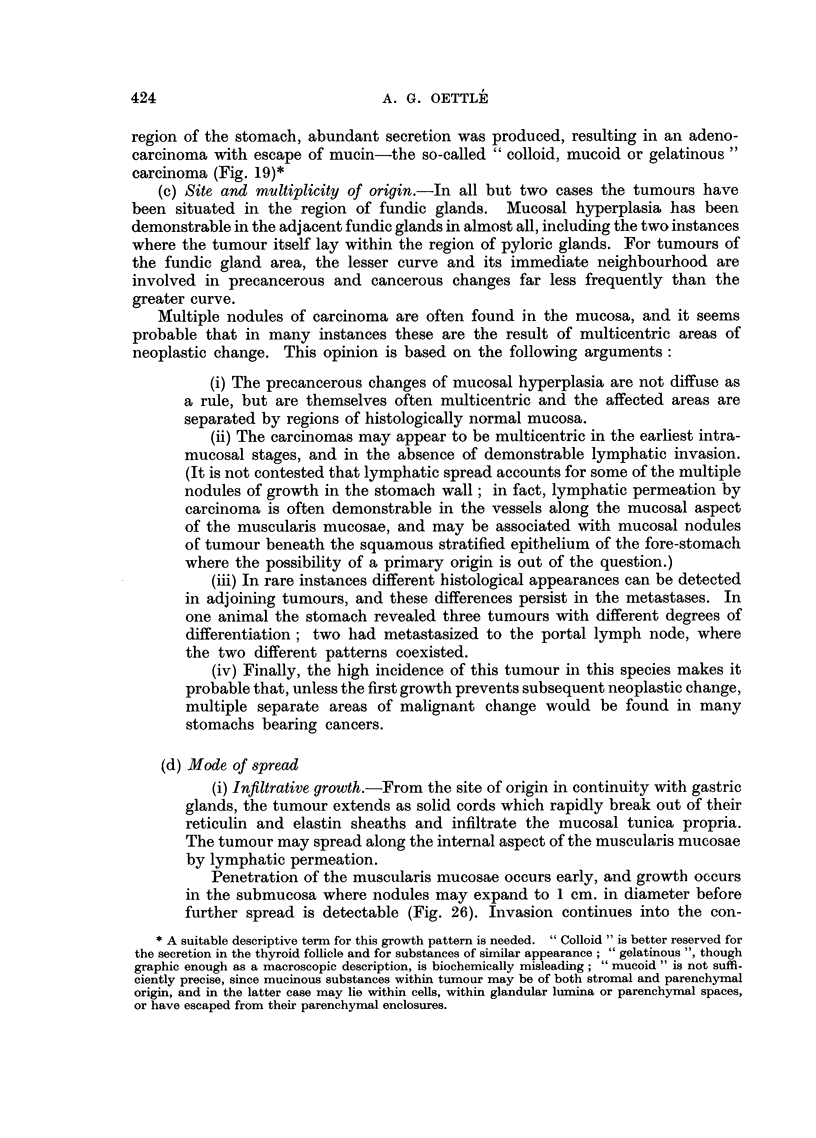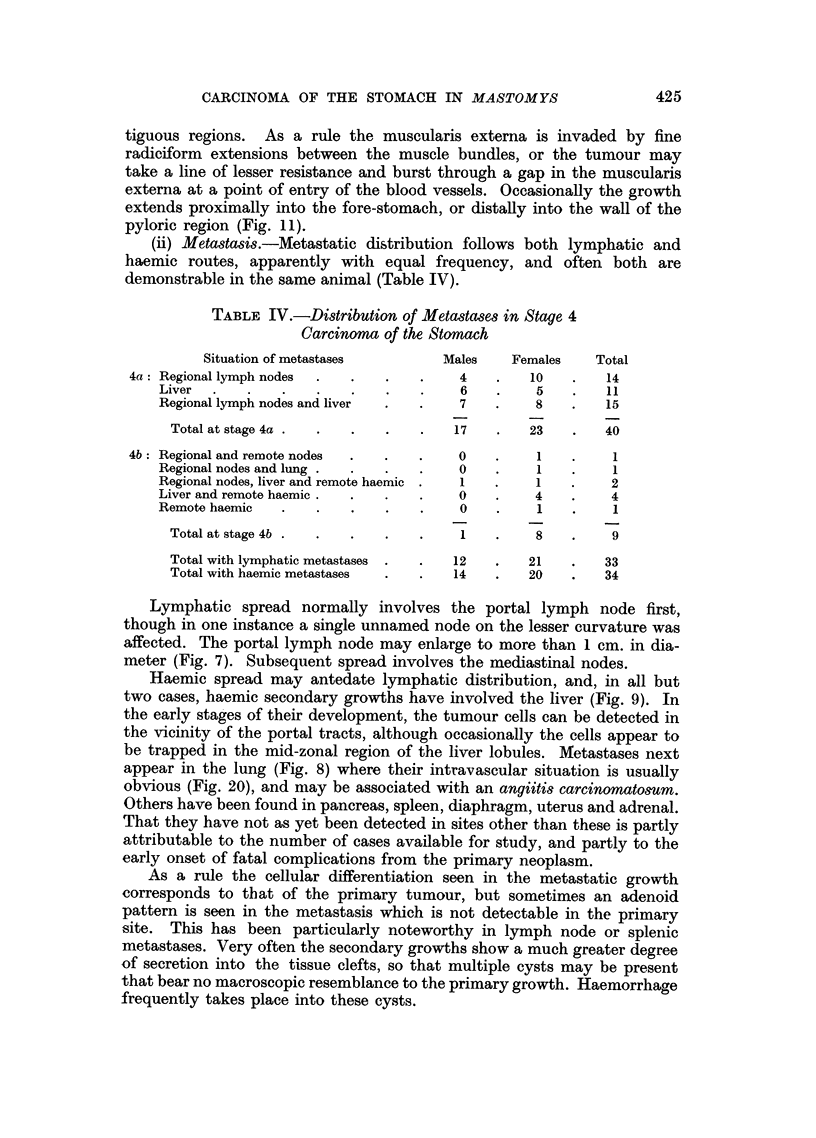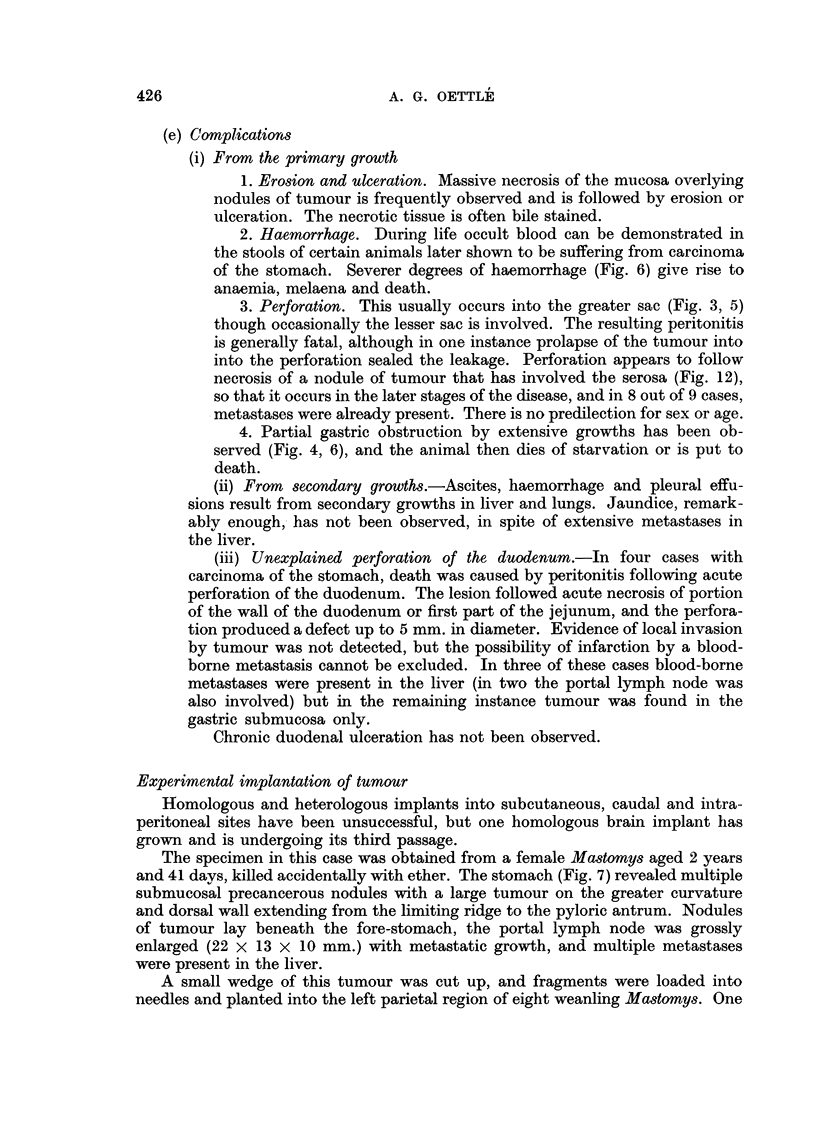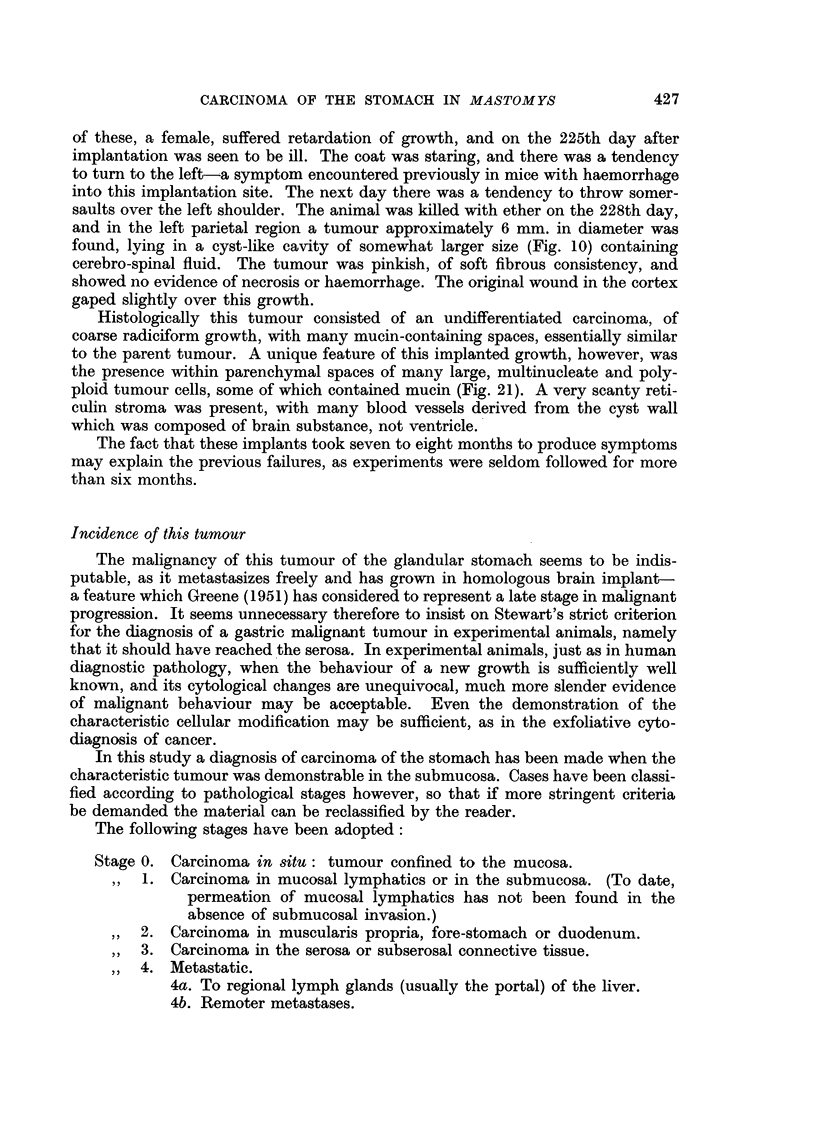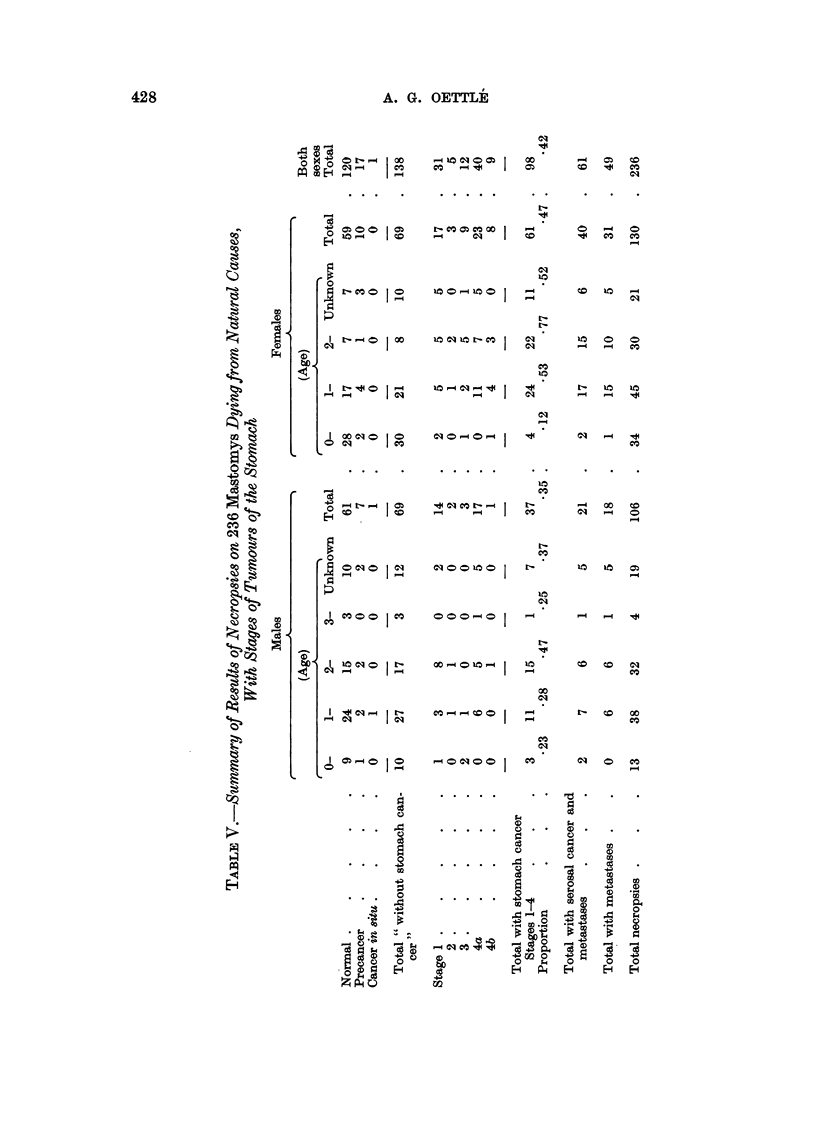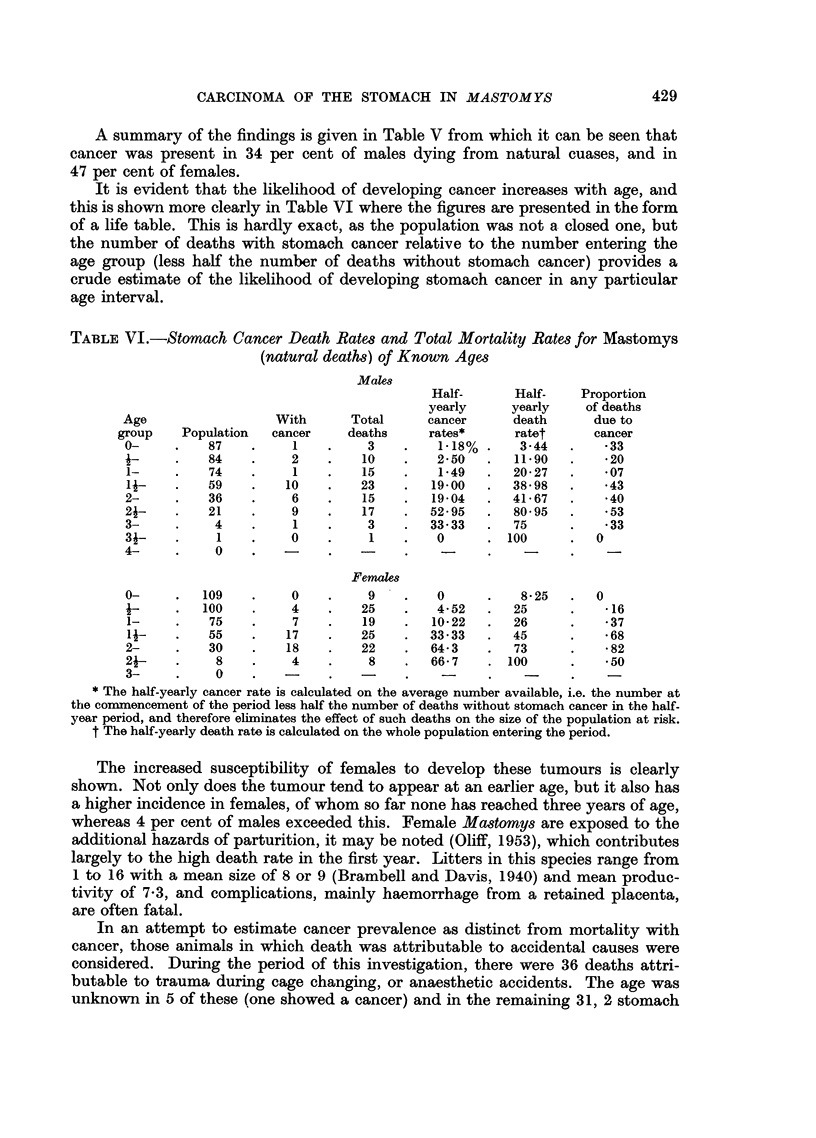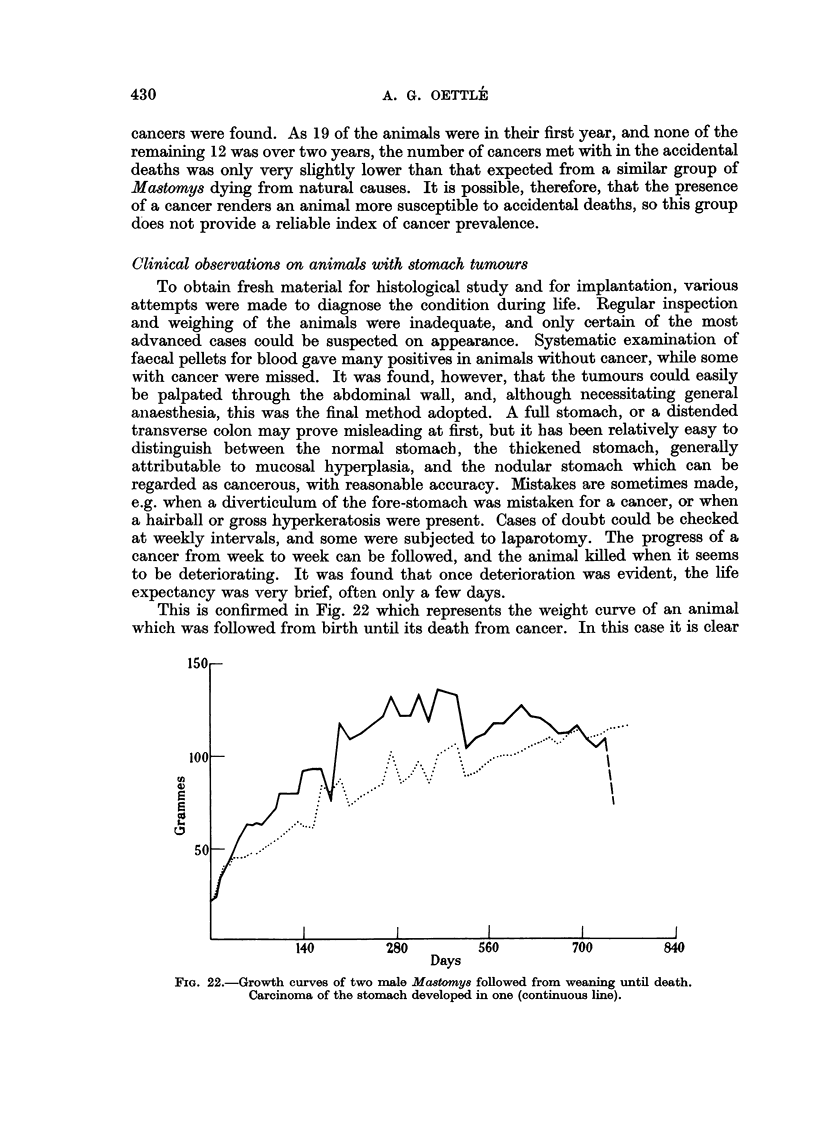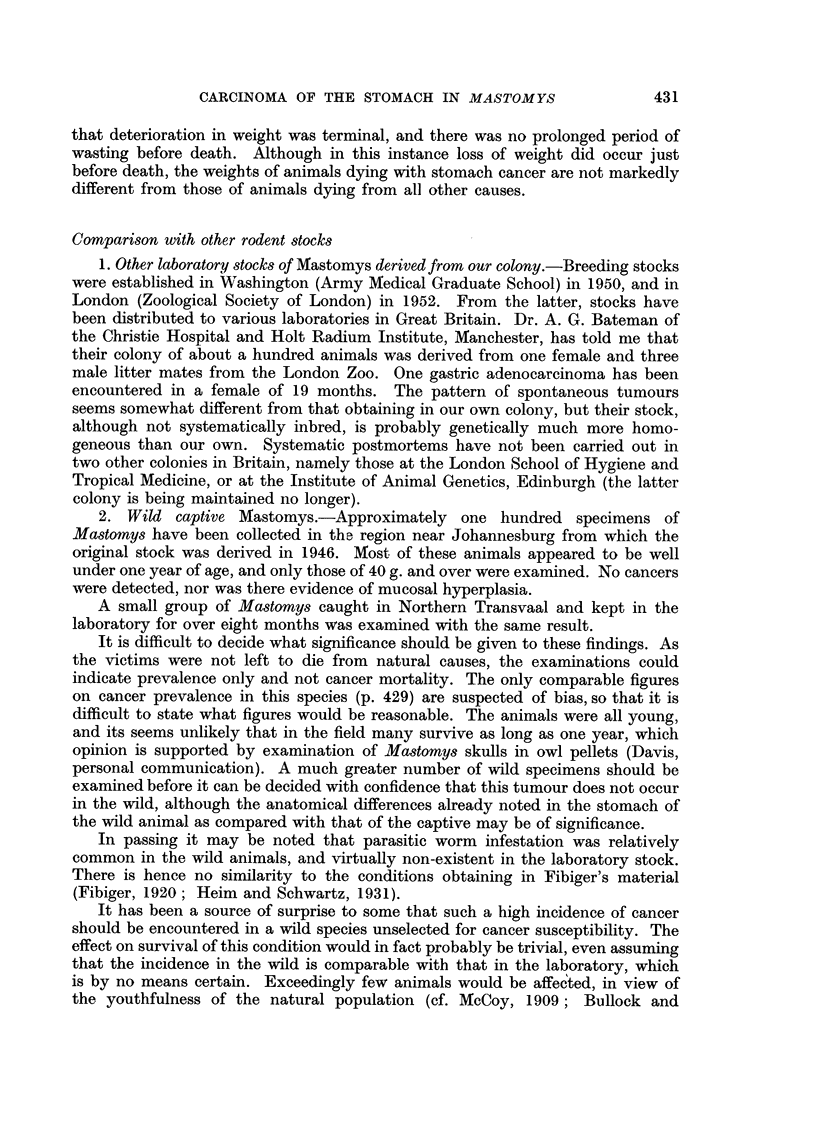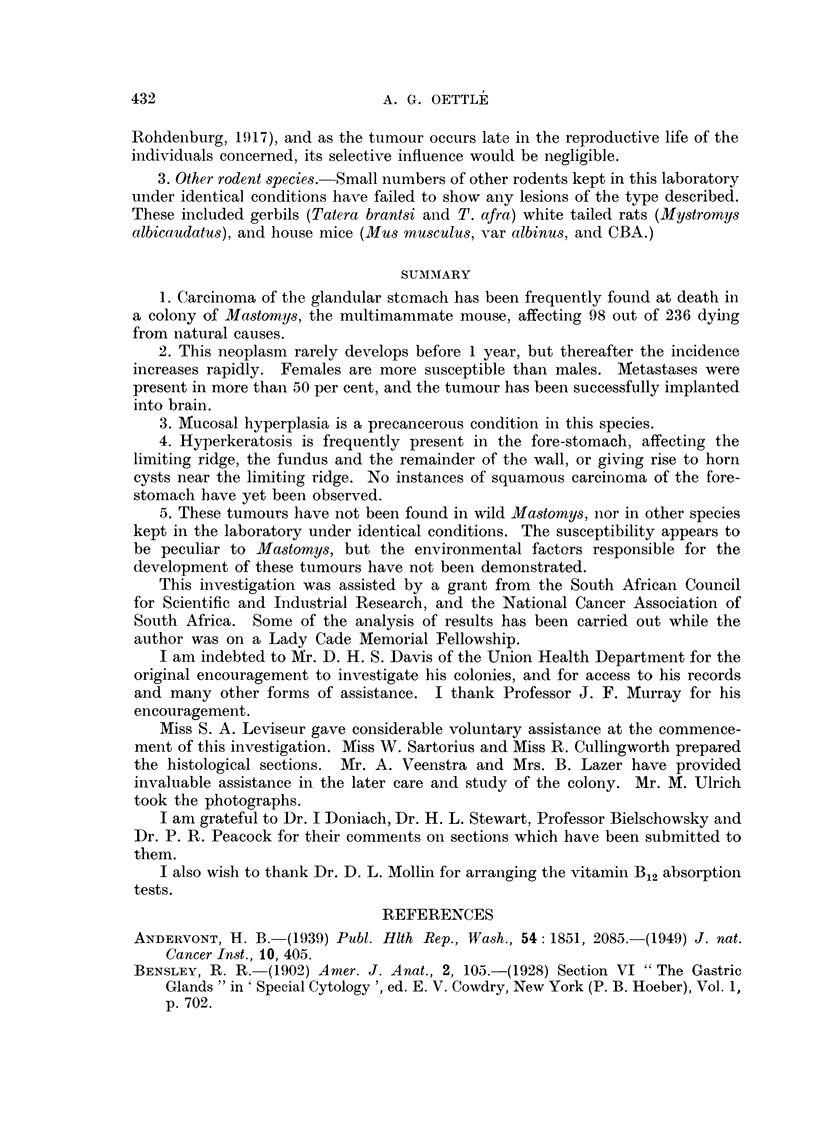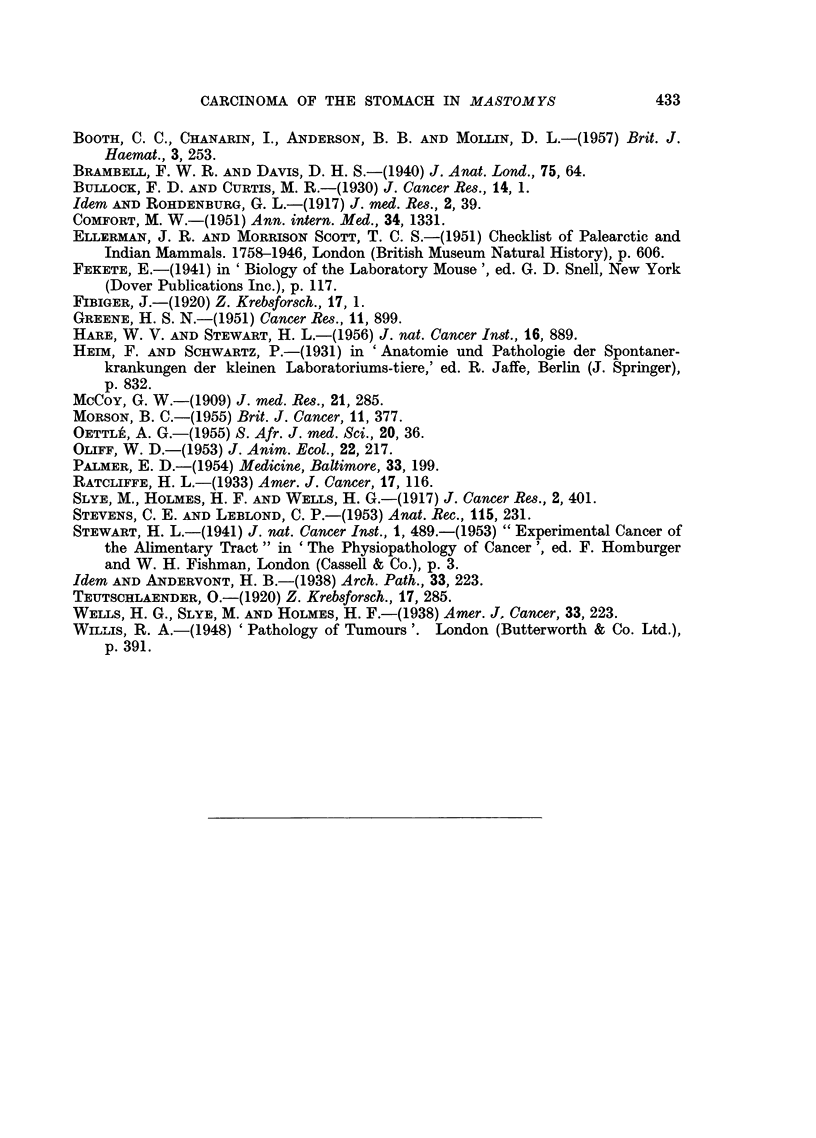# Spontaneous Carcinoma of the Glandular Stomach in Rattus (Mastomys) Natalensis, an African Rodent

**DOI:** 10.1038/bjc.1957.50

**Published:** 1957-09

**Authors:** A. G. Oettlé

## Abstract

**Images:**


### SPONTANEOUS CARCINOMA OF THE GLANDULAR STOMACH IN *RATTUS (MASTOMYS) NATALENSIS*, AN AFRICAN RODENT

A. G. OETTLÉ*

*From the South African Institute for Medical Research, Hospital Street, Johannesburg, South Africa*

* Lady Cade Memorial Fellow, National Cancer Association of South Africa.

Received for publication July 16, 1957

IN February 1954, Mr. D. H. S. Davis of the Union Health Department Plague Research Laboratory at this Institute asked me to investigate the causes of death in his colony of multimammate mice (*Rattus* (*Mastomys*) *natalensis*, A. Smith 1834, syn. *Mastomys coucha*) (Fig. 1). A previous life history study of this species (Oliff, 1953) had not led us to expect any unusual susceptibility to spontaneous tumours, and it was therefore surprising to find a carcinoma of the glandular stomach in the first necropsy, on a female between one and two years of age.

Subsequent investigations have shown that cancer of the stomach is remarkably frequent in this colony (Oettlé, 1955), although otherwise one of the rarest tumours in the lower animals (Slye, Holmes and Wells, 1917 ; Teutschlaender, 1920 ; Stewart, 1953) : in man it is usually a common cancer and in certain countries may account for more than 50 per cent of all malignant diseases (Willis, 1948). As regards laboratory rodents, Bullock and Curtis (1930) recorded 9 gastric sarcomas and one adenocarcinoma of the stomach in 33,000 necropsies on rats, while in over 142,000 mice of the Slye stock dying from natural causes, Wells, Slye and Holmes (1938) found 8 squamous carcinomas, 1 squamous papilloma, 2 adenocarcinomas, 2 adenomas and 1 sarcoma affecting the stomach. (I have omitted one adenocarcinoma diagnosed in *Peromyscus* in this series.) Cancer of the stomach has been noted in captive wild rodents by Ratcliffe (1933) who reported 3 adenocarcinomas of the " pylorus ", (i.e., presumably, the glandular stomach) in Japanese waltzing mice, *Mus wagneri rotans*, now known as *Mus musculus molissinus*, according to Ellerman and Morrison Scott (1951). Ratcliffe unfortunately omitted to mention how many necropsies were performed on this particular species, but in the 67 *Muridae* examined the number of waltzing mice could not have exceeded 61. In *Mastomys*, however, spontaneous adenocarcinoma of the glandular stomach has been detected in over 40 per cent of animals dying from natural causes in our colony, and its susceptibility to this tumour appears to be unparalleled.

### MATERIALS AND METHODS

### *The colony*

*Rattus* (*Mastomys*) *natalensis* is a rodent intermediate in many respects between a mouse and a rat : its common name, " the multimammate mouse " assigns it to the smaller, its scientific name *Rattus natalensis* to the larger genus. It has a number of unique anatomical features in addition, however, and it is probably more reasonable to regard it as belonging to a distinct subgenus *Mastomys*, and this term will be employed throughout this paper. Adults weigh between 40 and 80 g., occasionally exceeding the latter figure, and a few males attain weights over 100 g. A more detailed description of this species and our colony will be published elsewhere.

When my observations commenced the Johannesburg stock, which started with 17 pairs of wild captives, had passed through about ten generations in the laboratory without deliberate inbreeding. Since 1946 there have been no wild additions to the breeding stock. Oliff (1953) calculated the life-table of the females in this stock : in his study, carcinoma of the stomach is not mentioned among the causes of death, but complete necropsies were not performed at that time (Davis, personal communication).

The colony has been maintained under the same conditions as other rat and mouse colonies at this Institute. Cages are of galvanised iron, lined with sawdust, and measuring either 6 × 6 × 12 in. (15 × 15 × 30 cm.) or 6 × 18 × 18 in. (15 × 45 × 45 cm.) The temperature of the animal house is maintained thermostatically at 72–76 F. (22–24·5 C.) but may rise above this in summer. The standard laboratory diet for small rodents consists of commercial cubes and water *ad lib.* with fresh carrot thrice weekly. The composition of the cubes has been modified slightly during the life of the colony, e.g. up to October 1953, 10 lb. of cod-liver oil was employed per batch of cubes in place of the 1 lb. of vitamin A powder employed at present. The mixture is made in 5 ton (1116 kg.) batches, and is probably subject to minor batch variations, although each batch is analysed for its content of essential nutrients by the South African Bureau of Standards. The composition with a representative analysis of one of these batches is given in Table I.

TABLE I.—*Composition of Food Pellets*

| *Ingredients*— | |
|---|---|
| Fish meal | 680 |
| Yellow mealie meal, i.e. maize meal | 4,563 |
| Bran | 720 |
| Carcass meal | 906 |
| Ground oats | 1,350 |
| Monkey-nut meal, i.e. groundnut, peanut | 942 |
| Lucerne meal, i.e. alfalfa | 108 |
| Yeast extract | 96 |
| Molasses | 675 |
| Common salt | 50 |
| Limestone (ground Umzimkulu marble chips) | 50 |
| Synthetic Vitamin A : | |
| Powder (80,000 i.u./gm.) | 1 |
| | 10,141 lb. |

| *Representative analysis of one batch*— | | |
|---|---|---|
| Protein | | 18·8 |
| Fibre | | 5·1 |
| Fat | | 6·76 |
| Ash | | 8·04 |
| Calcium | 1·75 | |
| Phosphorus | 1·32 | |
| Moisture | | 9·16 |
| Carbohydrate (by difference) | | 52·14 |
| | | 100·00 |

*Pathological technique*

As soon as possible after a death is discovered, the abdomen is opened and sufficient 10 per cent formalin injected into the lumen of the stomach to produce gentle distension. The duodenum and oesophagus are not ligated. A complete necropsy is then performed, and all viscera are examined, with the exception of the spinal cord. For the most part only those organs showing macroscopic evidence of disease are taken for histological study. Tissues are usually fixed in 10 per cent neutral formalin-saline (4 per cent formaldehyde), and the stomach when excised is also immersed in fixative.

Paraffin sections are cut at 4 $\mu$ and stained with haematoxylin and eosin, or, when necessary, with picro-Mallory, periodic acid-Schiff, Gomori's aldehyde fuchsin, phosphotungstic acid-haematoxylin, toluidine blue, or Gordon and Sweet's modification for silver impregnation of reticulin.

Necropsies are carried out on all but the most putrid carcasses : in a very small proportion the corpses had been eaten by cage-mates or putrefaction was so advanced that examination seemed unprofitable, and the cadaver was excluded from the series. In addition to animals dying from natural causes, cases of unnatural and accidental deaths have also been examined, but have been omitted from the tables.

*Clinical studies*

Cancer of the stomach has been detected in some animals during life by the demonstration of occult blood in the faecal pellets with the benzidine and amidopyrine tests, but a more direct and reliable method is that of regular abdominal palpation. At present this has required light general anaesthesia on account of the wildness of this species. A group over 1 year of age was chosen, and a fraction of these was examined every week. Where tumours were suspected or diagnosed the individual was re-examined at weekly intervals until death.

Anaesthesia was induced by pumping air with a Higginson's syringe through a wash-bottle containing ether into a standard filter funnel of clear glass, $4\frac{1}{2}$ in. in diameter, under which the animal is trapped.

At first a number of anaesthetic deaths occurred, which have been attributed to formation of peroxides in old anaesthetic ether stored in clear glass bottles with large air spaces. With smaller dark bottles, containing a roll of fine copper mesh, these accidents have been much rarer, although old ether is still employed.

A more extensive discussion of the problems of handling this animal is to be published.

### OBSERVATIONS

Malignant tumours were encountered in more than half of the deaths from natural causes. Carcinoma of the stomach (Fig. 2) was the commonest, but other tumours included thymoma, adrenal cortical adenoma, thyroid carcinoma, rhabdomyosarcoma, angiomatous meningioma, acanthoma of skin and vagina (some of these were malignant), breast carcinoma, malignant hepatoma, and haemangiomas. Only the carcinomas of the stomach will be dealt with here.

### *Normal Anatomy and Physiology of the Stomach*

The stomach closely resembles that of the mouse as described by Fekete (1941). In wild specimens the fore-stomach represents about two-thirds of the total gastric mucosa, and is usually filled with the fibrous vegetable matter of the diet but in the laboratory stock used in this investigation, on a diet of greatly reduced fibre content, the fore-stomach is considerably smaller and contributes about one-third of the area (Fig. 2, 3, 4 and 5). A " cardiac antrum " is present. The glandular stomach comprises a proximal region of fundic glands, and a distal region of pyloric glands, distributed as in the mouse, while at the limiting ridge the fundic glands are replaced by two or three rows of cardiac glands, as described by Bensley (1902) in the rat.

In the contracted stomach the mucosa forms longitudinal ridges which are especially prominent along the greater curve.

Microscopically the extent of the fundic glands along the lesser curve is variable though always considerably less than that along the greater curve. The surface cells resemble those of the rat, and stain intensely with mucicarmine, periodic acid-Schiff (Fig. 15) and aldehyde-fuchsin. They are metachromatic with toluidine blue, but this is abolished with alcohol : the metachromasia can be preserved somewhat patchily by taking the dried section direct to xylene before mounting. In *Mastomys*, mucous neck cells stain very faintly with all mucin methods and their light pink, foamy cytoplasm in periodic acid-Schiff preparations is characteristic (Fig. 15). Both forms of mucin are argyrophilic with the reticulin method employed after periodic acid oxidation (Fig. 17).

A systematic investigation of gastric secretory activity has yet to be undertaken, but preliminary investigations of gastric secretion collected by the Shay technique of pyloro-ligature, revealed the presence of free acid. In a normal animal, e.g., 17·6 milliequivalents of free acid per litre with 54·3 milliequivalents of total acid per litre were demonstrated (Freed, unpublished observations).

Presumptive evidence was obtained for the presence of normal amounts of intrinsic factor in four animals, two normal but for gastric hairballs, and two had gastric carcinomas. These animals absorbed from 52 to 65 per cent of an oral dose of 0·0025 $\mu$g. of radioactive vitamin $B_{12}$. Similar amounts are absorbed by normal rats (Booth, Chanarin, Anderson and Mollin, 1957).

### *Pathological Observations*

### *Precancerous changes*

In the vicinity of almost all gastric tumours, as well as in certain stomachs without detectable carcinoma, the mucosa of the fundic region shows a characteristic type of mucosal hyperplasia (Fig. 12, 13, 14, 15), which in *Mastomys* appears to be precancerous. This type of hyperplastic change is the only pathological change in some stomachs, in others is found in association with the earliest intramucosal stages of carcinoma *in situ*, as well as accompanying florid carcinoma.

These changes are similar to the precancerous alterations in the jejunal mucosa of mice fed emulsions of carcinogenic hydrocarbons (Stewart, 1953) and are not unlike the gastric adenomatous lesions described by Stewart and Andervont (1938) and Andervont (1939, 1949) in mice of Strain I, and by Hare and Stewart (1956) in mice of Strain DBA. The spontaneous gastric adenomatous lesions of these mouse strains differ from those of *Mastomys*, however, in that the lesions in mice are not precancerous, affect males earlier than females, are detectable in all mice above a certain age, and are symmetrical.

TABLE II.—*Association of Precancerous Changes with Carcinoma of the Stomach*

| | Mucosal hyperplasia | |
|---|---|---|
| | Present | Not demonstrated |
| Without cancer of the stomach :* | | |
| Males . . . . . | 8 | 61 |
| Females . . . . | 10 | 59 |
| Total . . . . | 18 (13%) | 120 |
| With cancer of the stomach : | | |
| Males . . . . . | 35 | 2 |
| Females . . . . | 60 | 1 |
| Total . . . . | 95 (97%) | 3† |

* Stomachs which were macroscopically normal were not always sectioned, so that submacroscopic lesions would have been missed.

† In these the section taken was unsuitable for demonstration of precancerous changes. In two it was remote from the primary neoplasm. In the third was a small snip from a specimen preserved for mounting. What little mucosa was present revealed slight hyperplasia.

In *Mastomys* the macroscopic appearances vary both in degree and in distribution, and four types can be distinguished.

(a) *Prominence of rugae.*—In these stomachs there is hypertrophy of the longitudinal rugae of the mucosa (Fig. 6) which persists even though the stomach be distended. Superficial erosions and gastric haemorrhages are not uncommon, and if present provide strong macroscopic evidence of mucosal hyperplasia.

(b) *Nodularity.*—Focal hypertrophy may produce irregular mucosal modules, sometimes within the longitudinal folds, but often completely isolated from one another by mucosa which appears normal both macroscopically and microscopically. Sometimes only one or two nodules may be present in an otherwise healthy stomach

(c) *Cobblestone mucosa.*—In rare instances nodular hyperplastic changes are diffuse, regular and unrelated to the natural folds of the mucosa, which is covered with multiple small elevations (Fig. 7).

(d) *Polyps.*—True gastric polyps are rare, and represent a further development of isolated nodularity. The polyps have a pedicle which contains a core of submucosa, like their counterparts in human pathology.

All these changes are minimal along the lesser curve. Acute erosions with bleeding occur in all forms of the lesion but chronic peptic ulceration has not been observed.

External evidence of the presence of mucosal hyperplasia may be present as prominent stellate veins beneath the serosa, with a suggestion of crazy-paving in their demarcation of the areas of muscularis externa. This appearance is also commonly found in cases of carcinoma. When the mucosal hyperplasia is pronounced increased thickness of the gastric wall is detectable on palpation, as Stewart (1941) has noted in the adenomatous lesion of Strain I mice.

Microscopically, epithelial changes predominate, and interstitial cellular infiltration is not common in the mucosa (Fig. 13). In the underlying submucosa foci of lymphocytes are often detectable, usually at the base of a mucosal fold.

The surface cells are taller and the cytoplasm is more basiphilic than normal (Fig. 13). Secretory activity is minimal, although some show a distinct theca. The nuclei lie at different levels, which produces a pseudostratified appearance (Fig. 14). The secreted mucin, when present, retains its normal intensity of staining (Fig. 15). As a result of proliferation of the surface cells cyst-like dilatations of the gastric foveolae may be a striking feature, as well as bleb-like elevations of the superficial epithelium over an oedematous tunica propria.

In the gastric glands there is an increase in mucous neck cells (Fig. 15), which extend into the depths, where normally they are never encountered. This produces striking alterations in the proportions of the cell types in the gastric mucosa. Parietal and zymogenic cells are occasionally found among the abundance of mucous neck cells, and in addition one sometimes encounters areas of undifferentiated cells with acidophilic cytoplasm, resembling those of the gastric carcinoma (and therefore regarded as carcinoma *in situ*). An increased number of mitotic figures is noticeable in the isthmic regions (Fig. 14), and branching of the gastric glands is very prominent (Fig. 16, 17).

These are features of hyperplasia of the gastric epithelium, in which proliferation occurs in surface and mucous neck cells (Bensley, 1928). This concept is supported by Stevens and Leblond (1953), who, using colchicine methods, concluded that in the gastric epithelium, " with very rare exceptions, mitoses were confined to the two mucus-secreting types of cells " (p. 237). These authors distinguished two areas in which mitosis takes place, namely the deeper part of the isthmus, in which surface cells are dividing, and the neck region, in which mucous neck cell divisions are found. In *Mastomys*, hyperplasia may affect one or other of these regions and cell types predominantly, which results in an overgrowth of foveolar or glandular elements.

*Other hyperplastic conditions*

(a) *Mucosal extension into the submucosa.*—In *Mastomys*, as in other rodents suffering from hypertrophic gastric changes, one occasionally finds regions where gastric foveoli and associated glands have invaded the submucosa. These regions are sometimes surrounded by muscularis mucosae, but usually this is incomplete, and may not be detectable at all. Such downgrowths are not explicable on obliquity of section, since this would result in increased thickness of all layers, whereas the glandular tissue is reduced about such foveolae, and the epithelium is predominantly of surface type. This locally invasive condition is similar to that described by Stewart (1941) and by Hare and Stewart (1956), and as no transitional stages have been found between this and gastric carcinoma, it does not appear to be premalignant in *Mastomys*.

(b) *Hyperplasia of cardiac glands.*—Minor degrees of hypertrophy are sometimes detected in the cardiac glands, which may be increased in number, or irregular, dilated and branching. This seems to be associated with hyperkeratosis of the limiting ridge of the fore-stomach, and does not invariably accompany hyperplasia of the fundic glands nor does it appear to be precancerous.

(c) *Hyperkeratosis of the fore-stomach.*—This condition is frequently observed and is apparently benign, since malignant degeneration with the development of squamous carcinoma of the fore-stomach has not been observed. The incidence of hyperkeratosis increases until the second year, when approximately one in every two stomachs displays it in some form. It tends to be commoner in males than in females, and in animals with stomach cancer. There is no obvious relationship with the stage of the cancer, so the association of the two conditions, if real, appears to be indirect.

Hyperkeratosis may affect different regions of the fore-stomach and the following pathological varieties can be distinguished :

(i) *Hyperkeratosis of the limiting ridge.*—This region of the fore-stomach shows varying degree of thickening (Fig. 2, 6, 7, 11), up to about 5 mm., being scalloped, or occasionally having claw-like projections, and may overhang the glandular mucosa. This thickening sometimes extends on to the cardiac antrum (Fig. 2, 7).

(ii) *Horn cysts.*—These are encountered less frequently, a few millimetres from the limiting ridge, in the form of flask-like invasions of the submucosa, and are filled with squamous, keratinised material (Fig. 11). They are distinctly visible from the serosal aspect, as rounded white elevations 3 or 4 mm. in diameter.

(iii) *Diffuse hyperkeratosis of the fore-stomach.*—In these cases there is patchy heaping up of the horny layers of the epithelium in loose masses up to 5 mm. in thickness. The texture is far looser than that noted at the limiting ridge.

(iv) *Hyperkeratosis of the fundus.*—In these instances the fundus, which is usually contracted, is filled with loose layers of keratin (Fig. 6) up to 10 mm. in thickness. This condition is frequently associated with penetration by ingested hairs, which can be found projecting from the keratin mass, and also lying beneath the epithelium in the tunica propria. It appears that *Mastomys* swallows large amounts of hair, which in the starving animals may fill the otherwise empty stomach.

(d) *Diverticulosis of the fore-stomach.*—In one three-year-old male, a diverticulum measuring $9 \times 7 \times 4$ mm. was present in the fore-stomach. This diverticulum was not associated with malignant change, although the lesion was misdiagnosed clinically as a gastric carcinoma.

(e) *Atrophic gastritis and intestinal metaplasia.*—The frequent association of atrophis gastritis (Comfort, 1951) and intestinal metaplasia (Morson, 1955) with human gastric carcinoma has often been noted. Atrophic changes have been met in regions of submucosal invasion, as observed earlier, but are not a striking feature in *Mastomys*. It may be worth remarking that as the atrophic changes of the human stomach affect gastric glands essentially, they do not preclude hyperplastic changes more superficially (Palmer, 1954). In this respect the difference between the precancerous condition in *Mastomys* and that in man is not necessarily of great significance.

Intestinal metaplasia, on the other hand, has not been observed in *Mastomys*.

*Carcinoma of the glandular stomach*

(a) *Macroscopic appearance.*—In many cases the earlier stages of this tumour are macroscopically indistinguishable from mucosal hyperplasia, and consist of a series of irregular nodules of varying sizes in the gastric mucosa, with minimal alteration in the serosa or submucosa (Fig. 4).

In most instances, however, submucosal nodules are clearly distinguishable (Fig. 2, 4, 7, 11), usually near the greater curvature and often extending on to the ventral or dorsal gastric walls or both. The nodule may expand into the lumen with little or no sign of its presence externally, or it may produce a thick hard disc of tumour, which is seen externally as a distinct depression (Fig. 2). Multiple nodules may coalesce in a cauliflower-like growth (Fig. 7) that may obstruct the lumen.

---

### EXPLANATION OF PLATES

FIG. 1.—Adult *Mastomys* : the size is intermediate between rat and mouse.

FIG. 2.—Ventral half, showing small, contracted fore-stomach, with hyperkeratosis of the limiting ridge and cardiac antrum. An indentation externally on the greater curve indicates the site of a cauliflower growth here. Another growth occurs on the ventral wall, and also on the dorsal wall (not shown). Superficial haemorrhages are present. (C1480.)

FIG. 3.—Internal aspect of stomach showing a large perforation on the ventral aspect near the greater curve, in the centre of a disc of tumour. Haemorrhagic areas are present. There is a minor degree of hyperkeratosis of the limiting ridge. (C1347.)

FIG. 4.—The internal aspect of the dorsal half of this stomach shows hyperkeratosis of the limiting ridge, and the major portion of the glandular stomach along the greater curve is filled with diffuse tumour growth, almost completely obstructing the pyloric antrum, into which it bulges. (C828.)

FIG. 5.—External aspect of the perforation depicted in Fig. 3.

FIG. 6.—Fundal hyperkeratosis of the fore-stomach, with a few embedded hairs, prominence of longitudinal rugae in mucosa, and diffuse adenocarcinoma of the pyloric region with haemorrhage. (C894.)

FIG. 7.—Cobblestone mucosal hyperplasia with erosion in the ventral half of the stomach (middle specimen) and cauliflower growth on the dorsal wall in the fundic gland area (lower specimen). Mild hyperkeratosis of the limiting ridge and cardiac antrum is seen in both. The upper specimen is an enlarged portal lymph node. This tumour provided the successful brain implant. (C1416.)

FIG. 8.—Thoracic organs : a large thymoma is present above the heart, and multiple subpleural secondary nodules of a separate gastric carcinoma are visible in both lungs. (C490.)

FIG. 9.—Left dorso-lateral aspect of liver showing multiple solid metastases in the lateral, central and right upper lobes. (C1519.)

FIG. 10.—Brain showing in a cyst-space a large tumour implant ventral to the left lateral ventricle. (C1550.)

FIG. 11.—Fore-stomach with hyperkeratosis of the limitin ridge and three horn cysts. A large subserosal tumour and a smaller submucosal growth is evident. Necrosis of the mucosa is evident at the junction of these. Haemalum-eosin. × **4.**

FIG. 12.—Section through perforation through a submucosal tumour which had infiltrated the serosa. An overlying mucosal hyperplasia with erosion is evident. Haemalum-eosin. × 13.

FIG. 13.—Region of mucosal hyperplasia affecting gastric foveolae predominantly. (C169.) Haemalum-eosin. × 80.

FIG. 14.—Hyperplastic epithelium of foveola showing pseudostratification, mitotic figures, basiphilia and retention of secretory activity on the left side, as evidenced by the antra of the surface cells. Haemalum-eosin. × 375.

FIG. 15.—Region of isthmus in area of hyperplasia, showing mucin staining of antra in the foveola, and much paler mucin in mucus neck cells, which fill the tubules, apart from occasional parietal cells. Haemalum-periodic acid-Schiff. × 300.

FIG. 16.—Undifferentiated carcinoma with mitotic figures. Haemalum-eosin. × 375.

FIG. 17.—Reticulin framework of relatively undifferentiated growth. Mucin content of spaces in the tumour cords and nuclei have also been impregnated. Periodic acid-silver impregnation. × 75.

FIG. 18.—Portal lymph node containing an adenocarcinomatous metastasis from a stomach tumour. Haemalum-eosin. × 75.

FIG. 19.—Invasion of submucosa by adenocarcinoma with escape of mucin. Haemalum-eosin. × 75.

FIG. 20.—Solid cords of undifferentiated carcinoma within pulmonary arteries. Haemalum-eosin. × 95.

FIG. 21.—Implant into brain, demonstrating the scanty reticulin stroma, and that the multinucleate mucin-containing giant cells lie in spaces within the cords of undifferentiated tumour. (C1550.) Periodic acid-silver. × 300.

1

2

3

4

5

Oettlé.

6

7

8

9

10

11

12

13

Oettlé.

14

15

16

17

18

19

20

21

Less commonly the neoplasm spreads diffusely within the submucosa, and this thickening may extend widely over the stomach wall (Fig. 4), or be confined to the pyloric region (Fig. 6).

(b) *Histology*.—The majority of these tumours are undifferentiated carcinomas (Fig. 16) with minimal mucus secretion. The simple epithelial arrangement of the gastric glands is lost, and solid cords of strikingly uniform polyhedral cells are produced. These cells have a moderate amount of cytoplasm, with a little affinity for stains as a rule, though rarely the cytoplasm is eosinophilic. Occasional polyploid nuclei are detectable : mitotic figures are usually scanty.

The cells form fine and coarse radiciform* masses, with a minimal amount of stroma, consisting of reticulin (Fig. 17) with scanty elastin fibres. A variable number of blood vessels is present ; occasionally the tumour is highly vascular.

The larger islets of tumour often possess central clefts containing a colloid substance that reacts with varying intensities to mucin stains (Fig. 17). The cells surrounding these clefts are usually undifferentiated, show no orientation and possess neither the antra of the surface cells nor the foamy intracytoplasmic mucin of the mucous neck cells. Sometimes P.A.S. positive granules are detectable in the adjoining cytoplasm. The space may also contain varying amounts of blood : phagocytosis of the blood cells has not been observed.

In fewer than 10 per cent of the tumours (Table III) a more highly differentiated type of growth is encountered (Fig. 18, 19). Most of these well differentiated adenocarcinomas consist of tubules lined by a simple epithelium, that generally does not secrete mucin. In one stance, where the tumour arose in the pyloric

TABLE III.—*Degree of Differentiation in Stomach Carcinomas*

| | Age in years | Undiff. | Mixed undiff. and well diff. | Well diff. | With escaped mucin | Total |
|---|---|---|---|---|---|---|
| Males | 0– | 3 | 0 | 0 | 0 | 3 |
| | 1– | 8 | 2 | 1 | 0 | 11 |
| | 2– | 14 | 1 | 0 | 0 | 15 |
| | 3– | 1 | 0 | 0 | 0 | 1 |
| | Unknown | 7 | 0 | 0 | 0 | 7 |
| | Total | 33 | 3 | 1 | 0 | 37 |
| Females | 0– | 4 | 0 | 0 | 0 | 4 |
| | 1– | 23 | 1 | 0 | 0 | 24 |
| | 2– | 20 | 1 | 1 | 0 | 22 |
| | Unknown | 9 | 1 | 0 | 1 | 11 |
| | Total | 56 | 3 | 1 | 1 | 61 |
| Total both sexes | | 89 | 6 | 2 | 1 | 98 |

Counting cases with multiple tumours of different degrees of differentiation as separate growths there were 9 well differentiated tumours to 104 undifferentiated, i.e., 8 per cent.

* The term " radiciform " is suggested to describe solid invasive cords of growth, whether these be coarse or fine. The coarse pattern corresponds to that usually described as " alveolar " by pathologists. This term is already employed in anatomy and histology to describe a socket or empty space, as in the jaw or lung, and its etymologically invalid pathological application to solid islets or columns of carcinoma can only be maintained in the absence of a suitable synonym. I have chosen the term " radiciform " in preference to the more elegant " radicular " as the latter possesses other anatomical and pathological connotations.

region of the stomach, abundant secretion was produced, resulting in an adenocarcinoma with escape of mucin—the so-called " colloid, mucoid or gelatinous " carcinoma (Fig. 19)*

(c) *Site and multiplicity of origin.*—In all but two cases the tumours have been situated in the region of fundic glands. Mucosal hyperplasia has been demonstrable in the adjacent fundic glands in almost all, including the two instances where the tumour itself lay within the region of pyloric glands. For tumours of the fundic gland area, the lesser curve and its immediate neighbourhood are involved in precancerous and cancerous changes far less frequently than the greater curve.

Multiple nodules of carcinoma are often found in the mucosa, and it seems probable that in many instances these are the result of multicentric areas of neoplastic change. This opinion is based on the following arguments :

(i) The precancerous changes of mucosal hyperplasia are not diffuse as a rule, but are themselves often multicentric and the affected areas are separated by regions of histologically normal mucosa.

(ii) The carcinomas may appear to be multicentric in the earliest intramucosal stages, and in the absence of demonstrable lymphatic invasion. (It is not contested that lymphatic spread accounts for some of the multiple nodules of growth in the stomach wall ; in fact, lymphatic permeation by carcinoma is often demonstrable in the vessels along the mucosal aspect of the muscularis mucosae, and may be associated with mucosal nodules of tumour beneath the squamous stratified epithelium of the fore-stomach where the possibility of a primary origin is out of the question.)

(iii) In rare instances different histological appearances can be detected in adjoining tumours, and these differences persist in the metastases. In one animal the stomach revealed three tumours with different degrees of differentiation ; two had metastasized to the portal lymph node, where the two different patterns coexisted.

(iv) Finally, the high incidence of this tumour in this species makes it probable that, unless the first growth prevents subsequent neoplastic change, multiple separate areas of malignant change would be found in many stomachs bearing cancers.

(d) *Mode of spread*

(i) *Infiltrative growth.*—From the site of origin in continuity with gastric glands, the tumour extends as solid cords which rapidly break out of their reticulin and elastin sheaths and infiltrate the mucosal tunica propria. The tumour may spread along the internal aspect of the muscularis mucosae by lymphatic permeation.

Penetration of the muscularis mucosae occurs early, and growth occurs in the submucosa where nodules may expand to 1 cm. in diameter before further spread is detectable (Fig. 26). Invasion continues into the con-

* A suitable descriptive term for this growth pattern is needed. " Colloid " is better reserved for the secretion in the thyroid follicle and for substances of similar appearance ; " gelatinous ", though graphic enough as a macroscopic description, is biochemically misleading ; " mucoid " is not sufficiently precise, since mucinous substances within tumour may be of both stromal and parenchymal origin, and in the latter case may lie within cells, within glandular lumina or parenchymal spaces, or have escaped from their parenchymal enclosures.

tiguous regions. As a rule the muscularis externa is invaded by fine radiciform extensions between the muscle bundles, or the tumour may take a line of lesser resistance and burst through a gap in the muscularis externa at a point of entry of the blood vessels. Occasionally the growth extends proximally into the fore-stomach, or distally into the wall of the pyloric region (Fig. 11).

(ii) *Metastasis.*—Metastatic distribution follows both lymphatic and haemic routes, apparently with equal frequency, and often both are demonstrable in the same animal (Table IV).

TABLE IV.—*Distribution of Metastases in Stage* 4 *Carcinoma of the Stomach*

| | Situation of metastases | Males | Females | Total |
|---|---|---|---|---|
| 4*a* : | Regional lymph nodes | 4 | 10 | 14 |
| | Liver | 6 | 5 | 11 |
| | Regional lymph nodes and liver | 7 | 8 | 15 |
| | Total at stage 4*a* | 17 | 23 | 40 |
| 4*b* : | Regional and remote nodes | 0 | 1 | 1 |
| | Regional nodes and lung | 0 | 1 | 1 |
| | Regional nodes, liver and remote haemic | 1 | 1 | 2 |
| | Liver and remote haemic | 0 | 4 | 4 |
| | Remote haemic | 0 | 1 | 1 |
| | Total at stage 4*b* | 1 | 8 | 9 |
| | Total with lymphatic metastases | 12 | 21 | 33 |
| | Total with haemic metastases | 14 | 20 | 34 |

Lymphatic spread normally involves the portal lymph node first, though in one instance a single unnamed node on the lesser curvature was affected. The portal lymph node may enlarge to more than 1 cm. in diameter (Fig. 7). Subsequent spread involves the mediastinal nodes.

Haemic spread may antedate lymphatic distribution, and, in all but two cases, haemic secondary growths have involved the liver (Fig. 9). In the early stages of their development, the tumour cells can be detected in the vicinity of the portal tracts, although occasionally the cells appear to be trapped in the mid-zonal region of the liver lobules. Metastases next appear in the lung (Fig. 8) where their intravascular situation is usually obvious (Fig. 20), and may be associated with an *angiitis carcinomatosum.* Others have been found in pancreas, spleen, diaphragm, uterus and adrenal. That they have not as yet been detected in sites other than these is partly attributable to the number of cases available for study, and partly to the early onset of fatal complications from the primary neoplasm.

As a rule the cellular differentiation seen in the metastatic growth corresponds to that of the primary tumour, but sometimes an adenoid pattern is seen in the metastasis which is not detectable in the primary site. This has been particularly noteworthy in lymph node or splenic metastases. Very often the secondary growths show a much greater degree of secretion into the tissue clefts, so that multiple cysts may be present that bear no macroscopic resemblance to the primary growth. Haemorrhage frequently takes place into these cysts.

(e) *Complications*

(i) *From the primary growth*

1. *Erosion and ulceration.* Massive necrosis of the mucosa overlying nodules of tumour is frequently observed and is followed by erosion or ulceration. The necrotic tissue is often bile stained.

2. *Haemorrhage.* During life occult blood can be demonstrated in the stools of certain animals later shown to be suffering from carcinoma of the stomach. Severer degrees of haemorrhage (Fig. 6) give rise to anaemia, melaena and death.

3. *Perforation.* This usually occurs into the greater sac (Fig. 3, 5) though occasionally the lesser sac is involved. The resulting peritonitis is generally fatal, although in one instance prolapse of the tumour into into the perforation sealed the leakage. Perforation appears to follow necrosis of a nodule of tumour that has involved the serosa (Fig. 12), so that it occurs in the later stages of the disease, and in 8 out of 9 cases, metastases were already present. There is no predilection for sex or age.

4. Partial gastric obstruction by extensive growths has been observed (Fig. 4, 6), and the animal then dies of starvation or is put to death.

(ii) *From secondary growths.*—Ascites, haemorrhage and pleural effusions result from secondary growths in liver and lungs. Jaundice, remarkably enough, has not been observed, in spite of extensive metastases in the liver.

(iii) *Unexplained perforation of the duodenum.*—In four cases with carcinoma of the stomach, death was caused by peritonitis following acute perforation of the duodenum. The lesion followed acute necrosis of portion of the wall of the duodenum or first part of the jejunum, and the perforation produced a defect up to 5 mm. in diameter. Evidence of local invasion by tumour was not detected, but the possibility of infarction by a blood-borne metastasis cannot be excluded. In three of these cases blood-borne metastases were present in the liver (in two the portal lymph node was also involved) but in the remaining instance tumour was found in the gastric submucosa only.

Chronic duodenal ulceration has not been observed.

*Experimental implantation of tumour*

Homologous and heterologous implants into subcutaneous, caudal and intraperitoneal sites have been unsuccessful, but one homologous brain implant has grown and is undergoing its third passage.

The specimen in this case was obtained from a female *Mastomys* aged 2 years and 41 days, killed accidentally with ether. The stomach (Fig. 7) revealed multiple submucosal precancerous nodules with a large tumour on the greater curvature and dorsal wall extending from the limiting ridge to the pyloric antrum. Nodules of tumour lay beneath the fore-stomach, the portal lymph node was grossly enlarged (22 $\times$ 13 $\times$ 10 mm.) with metastatic growth, and multiple metastases were present in the liver.

A small wedge of this tumour was cut up, and fragments were loaded into needles and planted into the left parietal region of eight weanling *Mastomys*. One of these, a female, suffered retardation of growth, and on the 225th day after implantation was seen to be ill. The coat was staring, and there was a tendency to turn to the left—a symptom encountered previously in mice with haemorrhage into this implantation site. The next day there was a tendency to throw somersaults over the left shoulder. The animal was killed with ether on the 228th day, and in the left parietal region a tumour approximately 6 mm. in diameter was found, lying in a cyst-like cavity of somewhat larger size (Fig. 10) containing cerebro-spinal fluid. The tumour was pinkish, of soft fibrous consistency, and showed no evidence of necrosis or haemorrhage. The original wound in the cortex gaped slightly over this growth.

Histologically this tumour consisted of an undifferentiated carcinoma, of coarse radiciform growth, with many mucin-containing spaces, essentially similar to the parent tumour. A unique feature of this implanted growth, however, was the presence within parenchymal spaces of many large, multinucleate and polyploid tumour cells, some of which contained mucin (Fig. 21). A very scanty reticulin stroma was present, with many blood vessels derived from the cyst wall which was composed of brain substance, not ventricle.

The fact that these implants took seven to eight months to produce symptoms may explain the previous failures, as experiments were seldom followed for more than six months.

*Incidence of this tumour*

The malignancy of this tumour of the glandular stomach seems to be indisputable, as it metastasizes freely and has grown in homologous brain implant—a feature which Greene (1951) has considered to represent a late stage in malignant progression. It seems unnecessary therefore to insist on Stewart's strict criterion for the diagnosis of a gastric malignant tumour in experimental animals, namely that it should have reached the serosa. In experimental animals, just as in human diagnostic pathology, when the behaviour of a new growth is sufficiently well known, and its cytological changes are unequivocal, much more slender evidence of malignant behaviour may be acceptable. Even the demonstration of the characteristic cellular modification may be sufficient, as in the exfoliative cytodiagnosis of cancer.

In this study a diagnosis of carcinoma of the stomach has been made when the characteristic tumour was demonstrable in the submucosa. Cases have been classified according to pathological stages however, so that if more stringent criteria be demanded the material can be reclassified by the reader.

The following stages have been adopted :

Stage 0. Carcinoma *in situ* : tumour confined to the mucosa.
,, 1. Carcinoma in mucosal lymphatics or in the submucosa. (To date, permeation of mucosal lymphatics has not been found in the absence of submucosal invasion.)
,, 2. Carcinoma in muscularis propria, fore-stomach or duodenum.
,, 3. Carcinoma in the serosa or subserosal connective tissue.
,, 4. Metastatic.
4*a*. To regional lymph glands (usually the portal) of the liver.
4*b*. Remoter metastases.

TABLE V.—*Summary of Results of Necropsies on* 236 Mastomys *Dying from Natural Causes, With Stages of Tumours of the Stomach*

| | Males (Age) 0– | Males (Age) 1– | Males (Age) 2– | Males (Age) 3– | Males (Age) Unknown | Males Total | Females (Age) 0– | Females (Age) 1– | Females (Age) 2– | Females (Age) Unknown | Females Total | Both sexes Total |
|---|---|---|---|---|---|---|---|---|---|---|---|---|
| Normal | 9 | 24 | 15 | 3 | 10 | 61 | 28 | 17 | 7 | 7 | 59 | 120 |
| Precancer | 1 | 2 | 2 | 0 | 2 | 7 | 2 | 4 | 1 | 3 | 10 | 17 |
| Cancer *in situ* | 0 | 1 | 0 | 0 | 0 | 1 | 0 | 0 | 0 | 0 | 0 | 1 |
| Total " without stomach cancer " | 10 | 27 | 17 | 3 | 12 | 69 | 30 | 21 | 8 | 10 | 69 | 138 |
| Stage 1 | 1 | 3 | 8 | 0 | 2 | 14 | 2 | 5 | 5 | 5 | 17 | 31 |
| 2 | 0 | 1 | 1 | 0 | 0 | 2 | 0 | 1 | 2 | 0 | 3 | 5 |
| 3 | 2 | 1 | 0 | 0 | 0 | 3 | 1 | 2 | 5 | 1 | 9 | 12 |
| 4*a* | 0 | 6 | 5 | 1 | 5 | 17 | 0 | 11 | 7 | 5 | 23 | 40 |
| 4*b* | 0 | 0 | 1 | 0 | 0 | 1 | 1 | 4 | 3 | 0 | 8 | 9 |
| Total with stomach cancer Stages 1–4 | 3 | 11 | 15 | 1 | 7 | 37 | 4 | 24 | 22 | 11 | 61 | 98 |
| Proportion | ·23 | ·28 | ·47 | ·25 | ·37 | ·35 | ·12 | ·53 | ·77 | ·52 | ·47 | ·42 |
| Total with serosal cancer and metastases | 2 | 7 | 6 | 1 | 5 | 21 | 2 | 17 | 15 | 6 | 40 | 61 |
| Total with metastases | 0 | 6 | 6 | 1 | 5 | 18 | 1 | 15 | 10 | 5 | 31 | 49 |
| Total necropsies | 13 | 38 | 32 | 4 | 19 | 106 | 34 | 45 | 30 | 21 | 130 | 236 |

A summary of the findings is given in Table V from which it can be seen that cancer was present in 34 per cent of males dying from natural cuases, and in 47 per cent of females.

It is evident that the likelihood of developing cancer increases with age, and this is shown more clearly in Table VI where the figures are presented in the form of a life table. This is hardly exact, as the population was not a closed one, but the number of deaths with stomach cancer relative to the number entering the age group (less half the number of deaths without stomach cancer) provides a crude estimate of the likelihood of developing stomach cancer in any particular age interval.

TABLE VI.—*Stomach Cancer Death Rates and Total Mortality Rates for* Mastomys *(natural deaths) of Known Ages*

| Age group | Population | With cancer | Total deaths | Half-yearly cancer rates* | Half-yearly death rate† | Proportion of deaths due to cancer |
|---|---|---|---|---|---|---|
| | | | *Males* | | | |
| 0– | 87 | 1 | 3 | 1·18% | 3·44 | ·33 |
| ½– | 84 | 2 | 10 | 2·50 | 11·90 | ·20 |
| 1– | 74 | 1 | 15 | 1·49 | 20·27 | ·07 |
| 1½– | 59 | 10 | 23 | 19·00 | 38·98 | ·43 |
| 2– | 36 | 6 | 15 | 19·04 | 41·67 | ·40 |
| 2½– | 21 | 9 | 17 | 52·95 | 80·95 | ·53 |
| 3– | 4 | 1 | 3 | 33·33 | 75 | ·33 |
| 3½– | 1 | 0 | 1 | 0 | 100 | 0 |
| 4– | 0 | — | — | — | — | — |
| | | | *Females* | | | |
| 0– | 109 | 0 | 9 | 0 | 8·25 | 0 |
| ½– | 100 | 4 | 25 | 4·52 | 25 | ·16 |
| 1– | 75 | 7 | 19 | 10·22 | 26 | ·37 |
| 1½– | 55 | 17 | 25 | 33·33 | 45 | ·68 |
| 2– | 30 | 18 | 22 | 64·3 | 73 | ·82 |
| 2½– | 8 | 4 | 8 | 66·7 | 100 | ·50 |
| 3– | 0 | — | — | — | — | — |

* The half-yearly cancer rate is calculated on the average number available, i.e. the number at the commencement of the period less half the number of deaths without stomach cancer in the half-year period, and therefore eliminates the effect of such deaths on the size of the population at risk.

† The half-yearly death rate is calculated on the whole population entering the period.

The increased susceptibility of females to develop these tumours is clearly shown. Not only does the tumour tend to appear at an earlier age, but it also has a higher incidence in females, of whom so far none has reached three years of age, whereas 4 per cent of males exceeded this. Female *Mastomys* are exposed to the additional hazards of parturition, it may be noted (Oliff, 1953), which contributes largely to the high death rate in the first year. Litters in this species range from 1 to 16 with a mean size of 8 or 9 (Brambell and Davis, 1940) and mean productivity of 7·3, and complications, mainly haemorrhage from a retained placenta, are often fatal.

In an attempt to estimate cancer prevalence as distinct from mortality with cancer, those animals in which death was attributable to accidental causes were considered. During the period of this investigation, there were 36 deaths attributable to trauma during cage changing, or anaesthetic accidents. The age was unknown in 5 of these (one showed a cancer) and in the remaining 31, 2 stomach cancers were found. As 19 of the animals were in their first year, and none of the remaining 12 was over two years, the number of cancers met with in the accidental deaths was only very slightly lower than that expected from a similar group of *Mastomys* dying from natural causes. It is possible, therefore, that the presence of a cancer renders an animal more susceptible to accidental deaths, so this group does not provide a reliable index of cancer prevalence.

*Clinical observations on animals with stomach tumours*

To obtain fresh material for histological study and for implantation, various attempts were made to diagnose the condition during life. Regular inspection and weighing of the animals were inadequate, and only certain of the most advanced cases could be suspected on appearance. Systematic examination of faecal pellets for blood gave many positives in animals without cancer, while some with cancer were missed. It was found, however, that the tumours could easily be palpated through the abdominal wall, and, although necessitating general anaesthesia, this was the final method adopted. A full stomach, or a distended transverse colon may prove misleading at first, but it has been relatively easy to distinguish between the normal stomach, the thickened stomach, generally attributable to mucosal hyperplasia, and the nodular stomach which can be regarded as cancerous, with reasonable accuracy. Mistakes are sometimes made, e.g. when a diverticulum of the fore-stomach was mistaken for a cancer, or when a hairball or gross hyperkeratosis were present. Cases of doubt could be checked at weekly intervals, and some were subjected to laparotomy. The progress of a cancer from week to week can be followed, and the animal killed when it seems to be deteriorating. It was found that once deterioration was evident, the life expectancy was very brief, often only a few days.

This is confirmed in Fig. 22 which represents the weight curve of an animal which was followed from birth until its death from cancer. In this case it is clear

FIG. 22.—Growth curves of two male *Mastomys* followed from weaning until death. Carcinoma of the stomach developed in one (continuous line).

that deterioration in weight was terminal, and there was no prolonged period of wasting before death. Although in this instance loss of weight did occur just before death, the weights of animals dying with stomach cancer are not markedly different from those of animals dying from all other causes.

*Comparison with other rodent stocks*

1. *Other laboratory stocks of* Mastomys *derived from our colony.*—Breeding stocks were established in Washington (Army Medical Graduate School) in 1950, and in London (Zoological Society of London) in 1952. From the latter, stocks have been distributed to various laboratories in Great Britain. Dr. A. G. Bateman of the Christie Hospital and Holt Radium Institute, Manchester, has told me that their colony of about a hundred animals was derived from one female and three male litter mates from the London Zoo. One gastric adenocarcinoma has been encountered in a female of 19 months. The pattern of spontaneous tumours seems somewhat different from that obtaining in our own colony, but their stock, although not systematically inbred, is probably genetically much more homogeneous than our own. Systematic postmortems have not been carried out in two other colonies in Britain, namely those at the London School of Hygiene and Tropical Medicine, or at the Institute of Animal Genetics, Edinburgh (the latter colony is being maintained no longer).

2. *Wild captive* Mastomys.—Approximately one hundred specimens of *Mastomys* have been collected in the region near Johannesburg from which the original stock was derived in 1946. Most of these animals appeared to be well under one year of age, and only those of 40 g. and over were examined. No cancers were detected, nor was there evidence of mucosal hyperplasia.

A small group of *Mastomys* caught in Northern Transvaal and kept in the laboratory for over eight months was examined with the same result.

It is difficult to decide what significance should be given to these findings. As the victims were not left to die from natural causes, the examinations could indicate prevalence only and not cancer mortality. The only comparable figures on cancer prevalence in this species (p. 429) are suspected of bias, so that it is difficult to state what figures would be reasonable. The animals were all young, and its seems unlikely that in the field many survive as long as one year, which opinion is supported by examination of *Mastomys* skulls in owl pellets (Davis, personal communication). A much greater number of wild specimens should be examined before it can be decided with confidence that this tumour does not occur in the wild, although the anatomical differences already noted in the stomach of the wild animal as compared with that of the captive may be of significance.

In passing it may be noted that parasitic worm infestation was relatively common in the wild animals, and virtually non-existent in the laboratory stock. There is hence no similarity to the conditions obtaining in Fibiger's material (Fibiger, 1920 ; Heim and Schwartz, 1931).

It has been a source of surprise to some that such a high incidence of cancer should be encountered in a wild species unselected for cancer susceptibility. The effect on survival of this condition would in fact probably be trivial, even assuming that the incidence in the wild is comparable with that in the laboratory, which is by no means certain. Exceedingly few animals would be affected, in view of the youthfulness of the natural population (cf. McCoy, 1909 ; Bullock and

Rohdenburg, 1917), and as the tumour occurs late in the reproductive life of the individuals concerned, its selective influence would be negligible.

3. *Other rodent species.*—Small numbers of other rodents kept in this laboratory under identical conditions have failed to show any lesions of the type described. These included gerbils (*Tatera brantsi* and *T. afra*) white tailed rats (*Mystromys albicaudatus*), and house mice (*Mus musculus*, var *albinus*, and CBA.)

### SUMMARY

1. Carcinoma of the glandular stomach has been frequently found at death in a colony of *Mastomys*, the multimammate mouse, affecting 98 out of 236 dying from natural causes.

2. This neoplasm rarely develops before 1 year, but thereafter the incidence increases rapidly. Females are more susceptible than males. Metastases were present in more than 50 per cent, and the tumour has been successfully implanted into brain.

3. Mucosal hyperplasia is a precancerous condition in this species.

4. Hyperkeratosis is frequently present in the fore-stomach, affecting the limiting ridge, the fundus and the remainder of the wall, or giving rise to horn cysts near the limiting ridge. No instances of squamous carcinoma of the fore-stomach have yet been observed.

5. These tumours have not been found in wild *Mastomys*, nor in other species kept in the laboratory under identical conditions. The susceptibility appears to be peculiar to *Mastomys*, but the environmental factors responsible for the development of these tumours have not been demonstrated.

This investigation was assisted by a grant from the South African Council for Scientific and Industrial Research, and the National Cancer Association of South Africa. Some of the analysis of results has been carried out while the author was on a Lady Cade Memorial Fellowship.

I am indebted to Mr. D. H. S. Davis of the Union Health Department for the original encouragement to investigate his colonies, and for access to his records and many other forms of assistance. I thank Professor J. F. Murray for his encouragement.

Miss S. A. Leviseur gave considerable voluntary assistance at the commencement of this investigation. Miss W. Sartorius and Miss R. Cullingworth prepared the histological sections. Mr. A. Veenstra and Mrs. B. Lazer have provided invaluable assistance in the later care and study of the colony. Mr. M. Ulrich took the photographs.

I am grateful to Dr. I Doniach, Dr. H. L. Stewart, Professor Bielschowsky and Dr. P. R. Peacock for their comments on sections which have been submitted to them.

I also wish to thank Dr. D. L. Mollin for arranging the vitamin $B_{12}$ absorption tests.

### REFERENCES

ANDERVONT, H. B.—(1939) *Publ. Hlth Rep., Wash.*, **54** : 1851, 2085.—(1949) *J. nat. Cancer Inst.*, **10**, 405.

BENSLEY, R. R.—(1902) *Amer. J. Anat.*, **2**, 105.—(1928) Section VI " The Gastric Glands " in ' Special Cytology ', ed. E. V. Cowdry, New York (P. B. Hoeber), Vol. 1, p. 702.

BOOTH, C. C., CHANARIN, I., ANDERSON, B. B. AND MOLLIN, D. L.—(1957) *Brit. J. Haemat.*, **3**, 253.
BRAMBELL, F. W. R. AND DAVIS, D. H. S.—(1940) *J. Anat. Lond.*, **75**, 64.
BULLOCK, F. D. AND CURTIS, M. R.—(1930) *J. Cancer Res.*, **14**, 1.
*Idem* AND ROHDENBURG, G. L.—(1917) *J. med. Res.*, **2**, 39.
COMFORT, M. W.—(1951) *Ann. intern. Med.*, **34**, 1331.
ELLERMAN, J. R. AND MORRISON SCOTT, T. C. S.—(1951) Checklist of Palearctic and Indian Mammals. 1758–1946, London (British Museum Natural History), p. 606.
FEKETE, E.—(1941) in ' Biology of the Laboratory Mouse ', ed. G. D. Snell, New York (Dover Publications Inc.), p. 117.
FIBIGER, J.—(1920) *Z. Krebsforsch.*, **17**, 1.
GREENE, H. S. N.—(1951) *Cancer Res.*, **11**, 899.
HARE, W. V. AND STEWART, H. L.—(1956) *J. nat. Cancer Inst.*, **16**, 889.
HEIM, F. AND SCHWARTZ, P.—(1931) in ' Anatomie und Pathologie der Spontanerkrankungen der kleinen Laboratoriums-tiere,' ed. R. Jaffe, Berlin (J. Springer), p. 832.
MCCOY, G. W.—(1909) *J. med. Res.*, **21**, 285.
MORSON, B. C.—(1955) *Brit. J. Cancer*, **11**, 377.
OETTLÉ, A. G.—(1955) *S. Afr. J. med. Sci.*, **20**, 36.
OLIFF, W. D.—(1953) *J. Anim. Ecol.*, **22**, 217.
PALMER, E. D.—(1954) *Medicine, Baltimore*, **33**, 199.
RATCLIFFE, H. L.—(1933) *Amer. J. Cancer*, **17**, 116.
SLYE, M., HOLMES, H. F. AND WELLS, H. G.—(1917) *J. Cancer Res.*, **2**, 401.
STEVENS, C. E. AND LEBLOND, C. P.—(1953) *Anat. Rec.*, **115**, 231.
STEWART, H. L.—(1941) *J. nat. Cancer Inst.*, **1**, 489.—(1953) " Experimental Cancer of the Alimentary Tract " in ' The Physiopathology of Cancer ', ed. F. Homburger and W. H. Fishman, London (Cassell & Co.), p. 3.
*Idem* AND ANDERVONT, H. B.—(1938) *Arch. Path.*, **33**, 223.
TEUTSCHLAENDER, O.—(1920) *Z. Krebsforsch.*, **17**, 285.
WELLS, H. G., SLYE, M. AND HOLMES, H. F.—(1938) *Amer. J. Cancer*, **33**, 223.
WILLIS, R. A.—(1948) ' Pathology of Tumours '. London (Butterworth & Co. Ltd.), p. 391.